# Continuous and Periodic Expansion of CAG Repeats in Huntington's Disease R6/1 Mice

**DOI:** 10.1371/journal.pgen.1001242

**Published:** 2010-12-09

**Authors:** Linda Møllersen, Alexander D. Rowe, Elisabeth Larsen, Torbjørn Rognes, Arne Klungland

**Affiliations:** 1 Centre for Molecular Biology and Neuroscience, Institute of Medical Microbiology, Oslo University Hospital, Oslo, Norway; 2 Department of Informatics, University of Oslo, Oslo, Norway; 3 Institute of Basic Medical Sciences, University of Oslo, Oslo, Norway; Massachusetts General Hospital, United States of America

## Abstract

Huntington's disease (HD) is one of several neurodegenerative disorders caused by expansion of CAG repeats in a coding gene. Somatic CAG expansion rates in HD vary between organs, and the greatest instability is observed in the brain, correlating with neuropathology. The fundamental mechanisms of somatic CAG repeat instability are poorly understood, but locally formed secondary DNA structures generated during replication and/or repair are believed to underlie triplet repeat expansion. Recent studies in HD mice have demonstrated that mismatch repair (MMR) and base excision repair (BER) proteins are expansion inducing components in brain tissues. This study was designed to simultaneously investigate the rates and modes of expansion in different tissues of HD R6/1 mice in order to further understand the expansion mechanisms *in vivo*. We demonstrate continuous small expansions in most somatic tissues (exemplified by tail), which bear the signature of many short, probably single-repeat expansions and contractions occurring over time. In contrast, striatum and cortex display a dramatic—and apparently irreversible—periodic expansion. Expansion profiles displaying this kind of periodicity in the expansion process have not previously been reported. These *in vivo* findings imply that mechanistically distinct expansion processes occur in different tissues.

## Author Summary

Huntington's disease (HD) is a genetically determined neurodegenerative disorder identified by the presence of a mutation for a long series of CAG repeats (>36 repeats) in the Huntingtin (*HTT*) gene. Longer repeat sequences cause disease onset at a younger age. The mutation encodes an expanded glutamine tract within the huntingtin protein. This enlarged polyglutamine fragment in the protein leads to the formation of the huntingtin aggregates that are observed in HD brains. The stretch of CAG repeats expands with age in affected brain areas, increasing the length of the polyglutamine tract, and is believed to amplify the effect of the disease. Several HD mouse models display phenotypes relevant to the human disease. We have investigated the rate and modes of expansion in striatum, cortex, and tail in transgenic R6/1 mice. Tail was included as a stable tissue, however we observed a small continuous expansion of CAG repeats in tail tissues. In brain tissues, we identified a periodic expansion process consisting of predominantly seven repeat steps. Our findings point towards a very controlled molecular mechanism as the cause of expansion in the most severely affected tissues, which may provide useful targets that can be used to inhibit disease development.

## Introduction

Huntington's disease (HD) is a genetically determined neurodegenerative disorder, the onset of which is known to depend upon the length of glutamine-encoding CAG-repeat sequences lying within the Huntingtin *(HTT)* gene [1]. Humans may develop the disease if they have more than 36 repeats and disease onset usually starts during mid-life. An inverse relationship has been shown between CAG repeat length and age of onset in HD [2]–[5]. Additionally, somatic instability in human cortex has recently been shown to be a good predictor of disease onset [6]. Children with 108–256 CAG repeats are reported to show disease onset from one and a half years to six years of age [7].

Trinucleotide repeat (TNR) instability varies between organs in a variety of neurodegenerative disorders which are caused by expansion of CAG repeats in a coding gene, with the greatest instability observed in the brain [8]–[11]. In HD, striatum tissue shows the most severe neuropathology, followed by cortex. CAG length expansion is correlated with neuropathology and probably precedes the onset of symptoms [12]. The CAG repeat length is unstable in most cell types of the brain, but neurons tend to show the greatest mutation lengths in both humans and mice [13]–[15]. Meanwhile, minimal expansion is considered to occur in many other somatic tissues.

TNR sequences may form slipped strands during replication or repair, creating loops or hairpins, which protrude from the DNA duplex [16].

In the earliest model for repeat expansion the DNA polymerase forms slip-outs on the nascent strand leading to small-scale repeat expansion in repetitive sequences [17]. Loops of repeat-containing DNA are believed to cause either expansions or contractions during replication, when the slip-out occurs in the nascent or template strand, respectively [18], [19]. Several models have been suggested to explain TNR expansion during replication, such as folding of the lagging strand template into a hairpin, stalled replication forks and the orientation of the TNR in the genome, as well as the location of the origin of replication, as shown in several experiments in bacteria, yeast and human cells (Reviewed in [20]). More recently, a pertinent role of DNA repair proteins in CAG repeat expansion has been demonstrated *in vivo*. In particular, deletion of the mismatch repair (MMR) proteins, Msh2 and Msh3 [21]–[24] has been shown to abolish age-dependent somatic CAG repeat expansion in mouse models for HD. MMR has also been shown to be involved in TNR expansion in mouse models of myotonic dystrophy (DM1) [25]–[27]. Furthermore, the age-dependent expansion of TNR sequences in somatic cells was shown to be modified by the base excision repair (BER) 8-oxoguanine DNA glycosylase (Ogg1) in the R6/1 mouse model, demonstrating that there may be a link between oxidative DNA damage and TNR instability [13]. The flap endonuclease 1 (FEN1), which removes 5′-flaps during replication [28] and is involved in long-patch BER [29] is also implicated in expansion. Secondary TNR structures have been shown to inhibit FEN1 activity [30]. In addition to flap endonuclease activity, the EXO [31] and GAP activities of FEN1 have been shown to contribute to the resolution of TNR secondary structures *in vitro* [32]. Recently, it was shown that the stoichiometry of BER proteins, such as Ogg1, polymerase β and FEN1, may contribute to the tissue-selectivity of somatic HD CAG repeat expansion [33].

Nevertheless, the processes causing this expansion remain poorly understood, particularly in mammalian systems, although the formation of secondary DNA structures within the repeat sequence is thought to underlie the process [34]. Here we present evidence for two distinct modes of somatic expansion identified by the analysis of CAG repeat fragments from 103 HD R6/1 mice; a continuous slight expansion in tail, lung, heart and spleen, and a dramatic periodic expansion in striatum, and cortex, which we also compare to the expansions observed in liver. The continuous expansion process is shown here to conform to a bi-directional, forward-biased model that represents the occurrence of multiple short – tending towards unitary – CAG repeat insertions and deletions, at random moments as the mouse ages. In contrast, the dramatic expansion seen in brain tissues demonstrates a periodicity centred around seven repeats, which correlates with the stochastic insertion of stable TNR segments of consistent length. Meanwhile liver tissue shows a comparable average increase in CAG repeat length to striatum, but with a much weaker inclination to exhibit periodicity, tending more towards a continuum. This suggests either a much less controlled insertion length when compared to expansions in striatum, or that liver tissue undergoes both types of expansion simultaneously. We also present discursive models for these two expansion mechanisms. Identification of these two independent modes of expansion, in particular the tight mechanistic control implicit in the expansions within neuropathologically relevant tissues, increases our understanding of the tissue-dependent progress of HD. This brings us a step closer to inferring the *in vivo* mechanisms of the molecular components involved, by showing that only a limited selection of the existing models for expansion are able to explain the age-dependent CAG repeat expansions we observe.

## Results

### Fragment analysis shows tissue-specific modes of somatic CAG expansion

**Figure 1:**
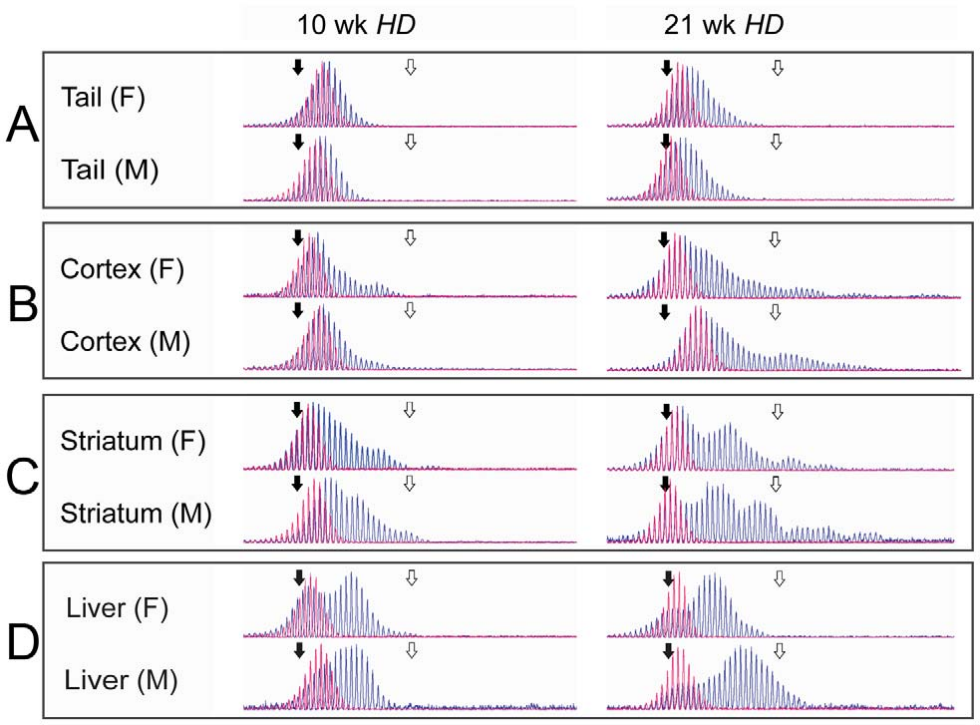
Fragment analysis in tail, cortex, striatum, and liver. Representative examples of raw data from CAG-repeat sequences in tail (A), cortex (B) striatum (C) and liver (D) from individual male and female HD mice, aged 10 and 21 weeks are shown (blue) with the tail biopsy from the same 3-week old mouse overlaid (red). All traces demonstrate the increase in mean length of repeat sequences with time and the differing rates of expansion between tissue types. Of particular note is the strong periodicity shown in the older striatum samples. Size standard markers are shown for 118 (solid black arrow) and 138 (open white arrow) CAG repeats respectively.

In order to understand the mechanisms underlying somatic CAG instability, 42 R6/1 HD exon 1 transgenic mice were sacrificed at either 10 or 21 weeks of age, whereupon tail, heart, lung, spleen, liver, cortex and striatum samples were taken for analysis of HD CAG repeat length. A tail biopsy at 3 weeks of age represents the reference level of CAG repeats present near birth in all tissues for each mouse [35] (Figure S3). Thus changes in the CAG composition of tissues in an individual mouse could be compared over a 7- or 18-week period. A slight expansion was observed in tail (Figure 1A), whereas cortex and striatum demonstrated a dramatic and periodic expansion process, with no significant difference between genders (Figure 1B and 1C). Liver demonstrated an equally rapid, but apparently more continuous expansion. Heart, lung and spleen displayed a slight expansion that was identical to tail (Figure S11). A parallel dataset from 61 *hHD^+/−^Fen1^E160D/E160D^* mutant mice, in which flap endonuclease activity of FEN1 is reduced to ∼20% [36] was included in the study. Reduced FEN1 endonuclease activity did not affect the rate of CAG repeat expansions measured in any tissues, implying that this mutation did not affect any role FEN1 plays in expansion. The two datasets were qualitatively and quantitatively identical with regard to the following analysis in all organs tested and were therefore combined, such that our analysis covers observations across two HD genotypes, reinforcing the ubiquity of the results.

### Continuous expansion of CAG repeats in tail

**Figure 2:**
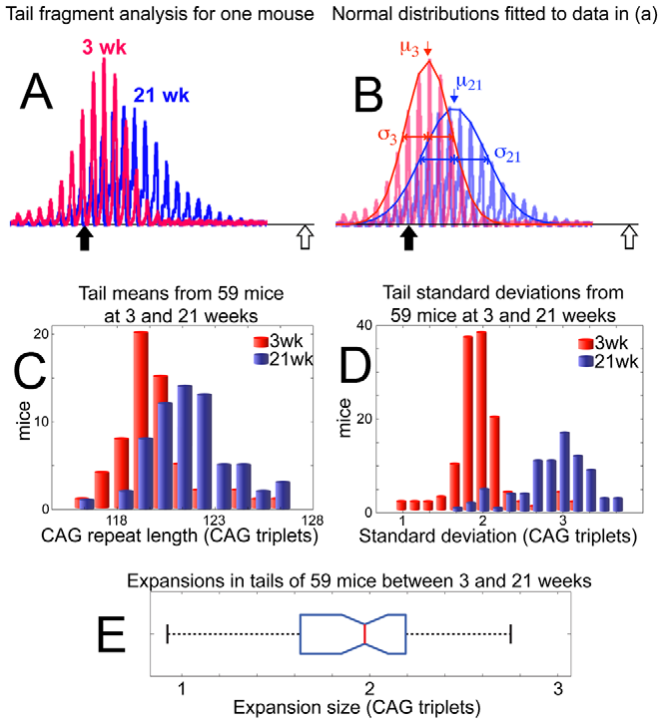
Slight continuous expansion measured in tail tissue. Size standard markers, are placed at 118 (solid black arrow) and 138 (open white arrow) CAG repeats. (A) A representative example of raw data from the 3-week biopsy (red) and 21-week sample (blue) are shown for one mouse. (B) Normal distributions are fitted to the data presented in (A), coloured as previously for 3-week (red) and 21-week (blue) samples. The resulting means (μ) and standard deviations (σ) are used to define the temporal change in repeat distribution within the sample, clearly demonstrating an increase in the mean number of repeats between 3 weeks (μ_3_) and 21 weeks of age (μ_21_). Likewise, broadening of the distribution is evident from the increase in standard deviation at 3 weeks (σ_3_) to that at 21 weeks (σ_21_). (C) Mean values (μ) for repeat lengths from the 3-week (red) and 21-week (blue) tails samples of 59 mice are compiled into a histogram, showing the systematic increase in repeat length with age. (D) Standard deviations of all 59 tail samples at 3-weeks (red) and 21-weeks (blue) are similarly compiled, with the histogram showing age dependent peak broadening. (E) A boxplot of expansions measured in the tails of 59 mice between 3 and 21 weeks of age shows a median expansion of 1.97 CAG repeats.

**Figure 3:**
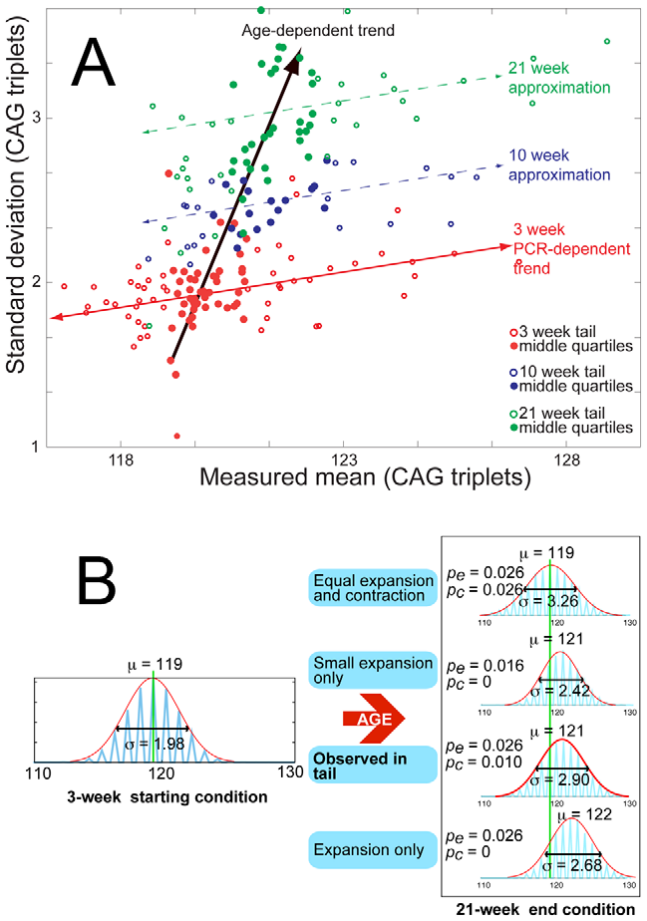
Age-dependent increase in mean and standard deviation for tail tissue. (A) Mean versus standard deviation points are plotted for all 103 mice in the data set, with the data divided into 3-week (red), 10-week (blue) and 21-week (green) age groups. To highlight the general trends in the data, the points representing the middle quartiles (by mean value) for each age group are shown with solid circles. The 3-week age group is shown with the weak positively-correlated trend line (red) implying a loose relation between standard deviation and mean value at a fixed age, which we assume to be caused by polymerase errors – during PCR of repeat sequences – which increase with sequence length. This trait is also present in 10-week and 21-week data, with parallel approximate trends shown (dashed) for emphasis. The standard deviations and means for each age group are, however, shown to increase systematically with age, along a trend-line (black arrow) that is completely separate from the PCR-dependent trend. This demonstrates the independence of the age-dependent increase in standard deviation and mean from the PCR induced variation. (B) Monte Carlo simulation of the proposed model mechanism for expansion is shown with a range of parameters for expansion and contraction probabilities *p_e_* and *p_c_*, to illustrate the change in mean and standard deviation of a distribution from a given starting point, dependent upon the relative expansion and contraction probabilities. It is clear from the results that only the combination of probabilities calculated from tail data can combine to generate the measured simultaneous change in mean and standard deviation.

Individual fragments from tail fit well to a normal distribution and are thus described by the mean (μ) and standard deviation (σ) of the curves fitted to raw data (Figure 2A, 2B; see Methods). Expansion within the population of 59 mice (10 week old mice excluded) is clearly shown (Figure 2C) by the relative difference in μ of the 21-week and 3-week groups. The median expansion found in 59 tails of 21-week mice was 1.97 CAG triplets (Figure 2E). In addition, σ of individual tail data sets is shown to increase from 1.98 triplets at 3-weeks to 2.87 triplets at 21-weeks (Figure 2D). The increasing σ is not an artefact of PCR errors, as is demonstrated in Figure 3A.

Having made these observations, it is necessary to consider them in the context of potential models for expansion, in order to fully investigate their implications and attempt to parameterize the processes involved. A continuous increase in both mean and standard deviation can be generally accounted for by multiple stochastic unitary (single CAG-repeats) extension and contraction events on the CAG tracts within the sample (Figure 3B). A full discussion of the potentially applicable models and considerations is presented as supporting information (Figure S8, Text S1, and Videos S1, S2, S3, S4, S5, S6, S7, S8) and we confine ourselves here to a simple application of the most probable hypothesis, yielding upper estimates for expansion and contraction rates. Assigning probabilities to non-simultaneous unitary expansions and contractions, 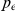 and 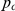 respectively, allows the measured temporal change in the mean (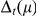) and variance (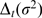) of tail samples to be defined by (1) and (2).
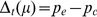
(1)

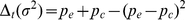
(2)Using a time interval (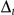) of one day, the measured expansion of 1.97 repeats and the concomitant increase in average standard deviation from 1.98 to 2.87, the values of 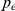 and 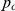 are calculated to be 0.026 and 0.010 respectively. This gives a maximum expectation of ∼0.036 (*pe*+*pc*) events per repeat tract per day.

### Periodic expansions in striatum and cortex

**Figure 4:**
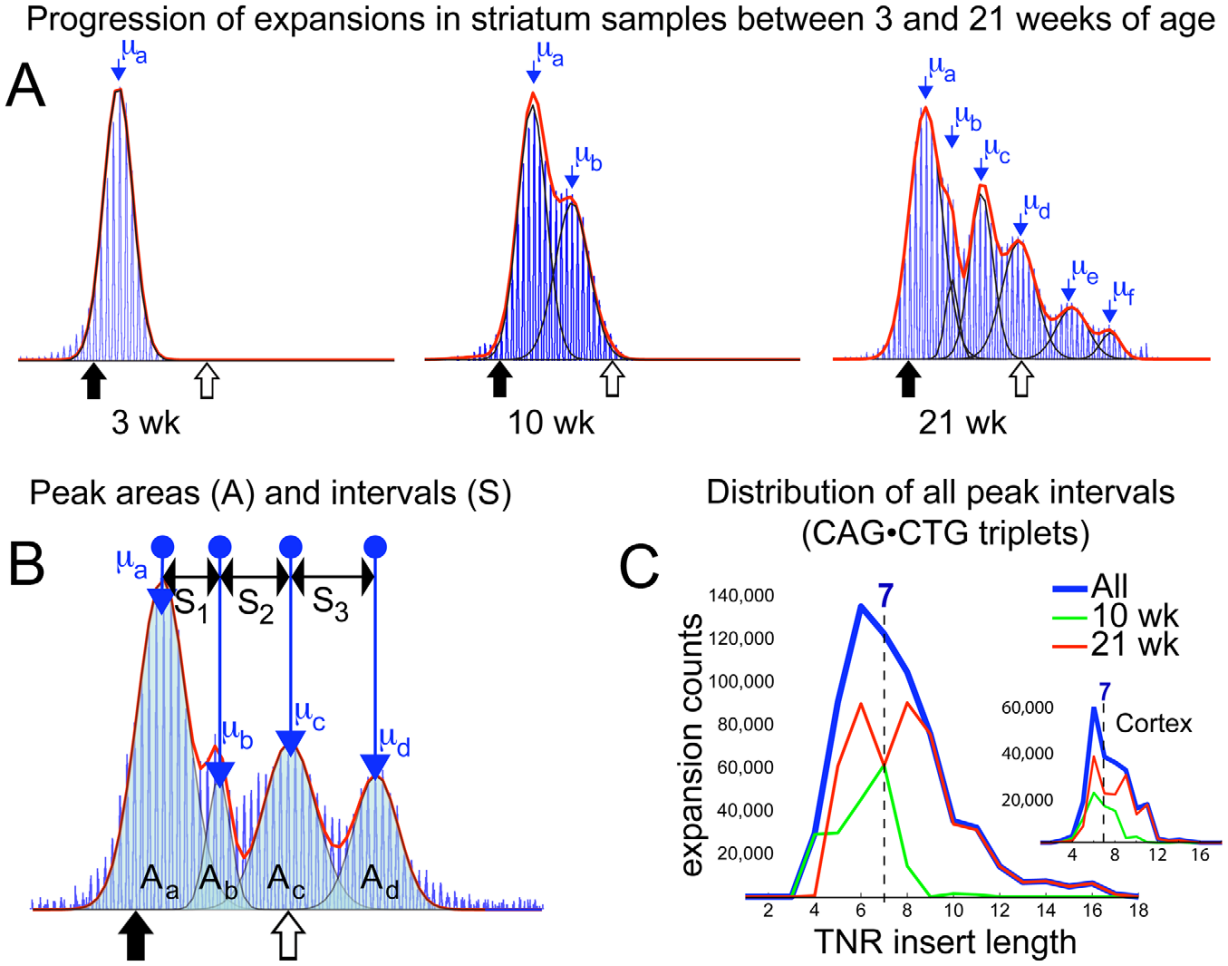
Periodic expansions in striatum and cortex. (A) Fragment analysis curves from 3-week tail, 10- and 21-week striatum are shown sequentially, with fitted normal distributions (black) overlaid. The sum of all fits (red) demonstrates the curve-fit accuracy. Mean values (μ_a_ etc) for each fitted peak are shown. Mean μ_a_ coincides with the mean of the corresponding 3-week tail at all ages. This 21-week striatum sample is best-fit by six consecutive normal distributions. Periodicity is clear from the regularity of the intervals between consecutive means. (B) Multiple peaks fitted to another 21-week striatum dataset are shown, with the areas under each fitted distribution (A_a_ .. A_d_), the mean values and the separations between the four peaks (S_1_ .. S_3_). The area of a peak represents the proportion of tissue containing the measured mean number of repeats, thus separation values represent a step-wise expansion from the previous mean. The age-dependent propagation of peaks with higher means, as seen in (A), is due to the stochastic insertion of short repeat sequences, of consistent length, into the CAG tracts of individual cells, which, over time, generates the observed periodicity. For analysis purposes, the sum of all areas (A_a,b,c,d_) was rescaled to 10,000 (the approximate number of cells forming a sample), allowing the number and length of insertion events to be estimated. (C) A histogram of insertion lengths for all expansion events measured in 69 separate striatum samples is shown (blue). Both mean and median values of the distribution point to a dominant insertion length of seven CAG repeats. The separate contributions from 10-week (green) and 21-week (red) data are also shown. Inset figure shows a similar result for cortex, with the insertion length distributed at 7 repeats, despite the smaller number of insertion events observed in total.

In contrast to fragment data from tail, heart, lung and spleen, raw data from 10- and 21-week cortex and striatum tissue show a peak retained at the 3-week repeat level alongside an age-dependent number of periodically spaced subsequent peaks (Figure 4A, Figures S1, S2, and S10), to which a series of normal distributions were fitted (see Methods). Knowing that the relative areas of overlapping distributions define the proportion of each mean CAG repeat length present (demonstrated with mixed samples and serial dilutions of a 21-week striatum sample in Figures S4 and S5), we infer – on account of the regularity of the intervals between neighbouring peaks at both 10- and 21-weeks of age – that expansion involves a proportion of the brain tissue undergoing insertions of consistent-length CAG repeat fragments over time. If expansion events inserted CAG fragments of uncontrolled length, the clarity of subsequent peaks would be lost. Likewise if different cell-types within one sample expanded at different rates, one would expect a continuum of peak separations in the collected accumulated data from many mice, and would have little reason to expect a consistent periodicity between peaks at 10-weeks and 21-weeks. This argues for the stochastic step-wise insertion of CAG fragments with an average length μ_b_−μ_a_ (Figure 4A (10wk and 21wk) and 4B) by a mechanism that may recur within the same cell. To measure the periodicity seen in brain tissues, we compiled histograms of all intervals between identifiable peaks, binned by size, from the individual cortex and striatum samples of mice aged 10- and 21-weeks (Figure 4C). The measured intervals are clearly shown to be distributed around a peak at 7 CAG repeats, with a mean length of 7.14 (σ = 1.78 with a cut-off for doublet measurements set at intervals ≥12). The median interval of 7 also confirms that this expansion process is centred around the insertion of 7-repeat fragments into the CAG tract, although the width of the starting distribution indicates insertion of 5 to 9 repeat-fragments (see Figure S9 and Videos S1, S2, S3, S4, S5, S6, S7, S8 for further discussion). The relative sizes of peaks in 21-week striatum (see Methods) imply that on average, a total of ∼10,000 7-repeat insertions occurred in each dramatically expanding striatal sample (on our timescale probably mainly within neurons; however, glial cells also undergo expansion and not all neuronal cells are guaranteed to show expansion [14], [15], giving a periodic expansion probability estimate 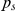 of 0.018 events per repeat tract per day. The fact that efficiency of PCR amplification of longer CAG tracts is reduced, may result in some measure of underestimation with this value. This is ∼70% of the estimated probability for unitary expansion events in tail, however the 7-repeat average insertion size renders the resulting expansion more dramatic in striatum. In contrast, liver data while showing comparable levels of average expansion to brain tissues, lacks a clear signature of periodicity (see Figure 1D) tending towards bimodality, with a more continuously located second peak. This would imply a much less controlled insertion length during the expansion process, or possibly a combination of expansion mechanisms.

## Discussion

Having shown data and analysis to define these two distinct modes of expansion, we place our findings into the context of existing literature, in order to develop reasonable hypothetical models for these two types of expansion.

The continuous expansion we observe shows a progressive increase in CAG tract length, which is comparable with the expansions observed in fibroblasts derived from an adult HD mouse [37]. In the debate regarding the relative roles of replication and repair in TNR instability, these results present an interesting question, since the continuous expansion process occurs in organs containing dividing cells. A process occurring with the previously calculated expectation rate of ∼0.036 events per repeat tract per day on the ∼360 nucleotide CAG segment of the 2.5 gigabase mouse genome, would correlate to ∼250,000 genome-wide events per cell per day. This is well above the upper estimate for daily DNA damage events, making it unlikely that these are the sole initiator of expansions in tail tissue. Several replication-based models for TNR expansion in dividing cells [18], [19] have been proposed. The potential for replication to be entirely responsible for this expansion is considered in detail elsewhere (Figures S7, S8, and Text S1) and is considered to be unlikely, particularly in light of the fact that lymphoma tissues (with necessarily higher replication rates) isolated from several mice showed no increase in TNR instability (Figure S7). Therefore we infer that other mechanisms must also prevail, and propose that slipped strand structures [16], [38] generated by out of register rehybridization of CAG repeats [27] during transcription, or genome maintenance, may spontaneously form unstable loops or cruciforms which may subsequently stabilize by migrating apart. Similar small loops can also be formed by polymerase slippage during replication [17]. We propose that two separate pathways may repair these loops, leading to single repeat expansions or contractions (Figure S8). Further research is needed to resolve the specific origin of this mode of expansion.

A hypothesis for periodic expansion is also presented (Figure S9). The previously calculated periodic expansion probability 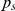 of 0.018 events per repeat tract per day would correlate to ∼100,000 genome-wide events per cell per day. This lies in the vicinity of upper estimates for accumulated oxidative DNA damage [39]. It is therefore possible that DNA damage contributes as catalyst for periodic expansions in brain tissues. However, oxidative damage is not sufficient to trigger somatic instability [33]. Of particular interest here, are the potential molecular components that could repeatedly generate a regularly sized repeat insertion that is dominantly seven repeats in length. We have therefore chosen to briefly review the relevant literature in search of further insight.

Theory suggests that CAG flaps ranging from 4 to 16 triplet repeats in length can form thermodynamically unstable hairpin structures with an even number of repeats [40]. However, under physiological conditions, 6 triplet repeats have been shown to form hairpins irreversibly [41], [42]. This implies that a progressively generated triplet repeat flap can stabilise into a 6-repeat hairpin at the free end that could be cleaved by FEN1 causing no net expansion [43] (Figure S9). However, the presence of metastable intermediates during flap generation may allow the flap length to increase beyond 6 repeats before a stable structure is formed, thereby producing a hairpin with an overhanging CAG at the 5′-end. This free CAG repeat can hybridise back to the DNA duplex, and it has been shown by Liu *et al.* [31] that such hybridisation facilitates bubble formation followed by ligation and expansion. In a few instances, two or even three free CAG repeats in the 5′-end of the hairpin would produce a periodicity of eight or nine CAG repeat steps. In addition, the size of the hairpin could vary with a few repeats. However, this occurs more rarely, as observed in Figure 4C. After gap filling and ligation, a loop of excess CAG repeats in one strand would be produced (Figure S9) that can be recognized by the MMR complex Msh2-Msh3 with high affinity [44]. This binding might further stabilize the CAG loop and additionally explain why Msh2-Msh3 is necessary for large expansions to occur in striatum [21]–[24], although the role of MMR in causing TNR expansion is not understood. Moreover, Msh2-Msh3 function ceases due to impaired ATPase activity on loops exceeding 16 nucleotides in lengths [45] - probably due to the presence of A•A mispaired bases in the loop [22] - which may explain why the MMR system fails to repair longer extraneous CAG loops. Subsequently, a nick generated on the strand opposite to the loop could result in faulty repair of the CTG strand along the CAG slip-out, causing expansion by a repair process independent of MMR [46] (Figure S9). Thus, oxidative damage on the CTG strand could result in base excision repair (BER) and Ogg1 moderated expansion, which is also in concordance with the proposed ‘toxic oxidation cycle’ by Kovtun et al., 2007 [13]. We therefore propose that a coincidental cooperation can occur between MMR and BER in cases where a long CAG flap is able to stabilize itself, which can form the basis of the consistent periodic insertion we observe.

It should be pointed out that it is possible that other stabilized structures, such as loop-outs or a stabilizing interaction with one of the many proteins and complexes that are in contact with the DNA may also serve to cause the observed periodicity. The majority of the potentially applicable models are covered in the literature [13], [24], [27], [34]. Further work is necessary to resolve this completely.

Cell proliferation in neurons and glial cells has been observed in the subependymal layer adjacent to the caudate nucleus in human HD brains [47]. However, polymerase slippage usually forms small expansions [17], and the repetitive uniformity of the periodic expansion makes it unlikely that polymerase slippage is responsible for the dramatic expansions seen in cortex and striatum. Furthermore, the lack of periodically spaced peaks containing fewer repeats than the mice were born with – as would be expected from a bi-directional process - means that 7-repeat hairpin-based contraction events occur either at a negligible rate in comparison to expansions, or do not occur at all. During the 18 week period, tail-type expansion events are not evident in striatum since they would obfuscate latter periodic peaks (Figure S6). Therefore, the two expansion mechanisms seem to be either entirely independent – not occurring simultaneously in the same cell – or that if they do share common elements, they progress along mutually exclusive pathways. However, it is important to notice that both expansion mechanisms must be dependent on proteins from the MMR complex, since expansions are eliminated in all tissues in either *Msh2* or *Msh3* nulls in HD [21]–[24], and also in DM1 [25], [26]. To date, we have not managed to define the individual cell types that are specific to these expansion mechanisms. It will be of great interest to compare the expansions observed in animal models to those in cultured HD mouse fibroblasts, as a way of identifying the cell specificity of these modes of expansion.

The liver may be particularly interesting in this regard, since this tissue potentially exhibits a mix of both types of expansion mechanisms. This could be attributed to different modes of expansion occurring in different cell types. Indeed, instability of the DM1 CTG•CAG repeat is known to occur in liver hepatocytes with high DNA ploidy [48].

The question also arises as to why FEN1 did not influence CAG repeat expansion in the organs tested. One might expect a difference, since a recent *in vitro* study has shown that FEN1, together with long-patch BER of long repeat sequences by polymerase β, promotes expansion by facilitating the ligation of hairpins formed by strand slippage [49]. However, FEN1 flap activity has shown to be circumstantial, with much lower activity in the striatum than in the cerebellum of R6/1 mice [33]. In yeast the capture of flap structures by FEN1, rather than the endonuclease activity, is the most important function of FEN1 in preventing TNR expansion [50]. EXO- and GAP activity of FEN1 are also involved in *in vitro* triplet repeat expansion in yeast [32], and these activities are probably not influenced by the *Fen1^E160D/E160D^* mutation. Therefore, it seems that the 20% endonuclease activity [36] of the *Fen1^E160D/E160D^* mutation does not affect the rate of CAG repeat expansions. In concordance with our finding Fen1 did not control instability of (CTG)_n_*(CAG)_n_ repeats in a knock-in mouse model for DM1 [51].

It is worth considering briefly why this periodicity has not been described before, since there are numerous potential reasons. One possibility could be that the mice used in the present study exhibit more instability due to environmental factors [52] or genetic background [53]. Perhaps the periodic signal becomes more disperse in older mice used elsewhere; degrading the quality of the data, and that the age-range, as well as the relatively long starting CAG lengths, of our samples is optimal for observing this periodicity. Another possibility is simply that later versions of the GeneMapper system are more sensitive, in comparison to the GeneScan method applied in some older studies, allowing us to see more detail. While periodicity has not featured in other studies of similar tissues and disease models, it is difficult to state unequivocally that it was unobservable in their data. The small volume of data presented in articles, uncertainty over the precise PCR conditions used and the simple fact that periodicity was not the focus of these investigations can be sufficient cause for this phenomenon to have been previously overlooked. There is some variability among replicate striatum samples as shown in Figure S10. This could be explained by sampling error or polymerase slippage in early PCR cycles. Sampling error is however unlikely to be the reason behind the periodicity as explained in Figure S12 and Text S2.

So far, we have only studied periodicity in the R6/1 mouse model and without specific studies of other HD CAG mice models the generality of our data is unknown. Yet, the R6/1 transgenic mouse is a widely accepted and commonly used model for human HD that exhibits a progressive neurological phenotype that exhibits many of the features of HD [54]. *HD* CAG repeat instability has shown to be similar in humans and mice, with the longest expansion lengths occurring in striatum, followed by cortex, and little expansion in cerebellum and most other tissues [8], [14], [35]. In addition, the *HD* CAG repeat length appears to be expand most in neuronal cells rather than glial cells in both species [14], [15]. Due to the long starting CAG repeat length in the transgenic mouse, the model may be most relevant as a model for juvenile HD. Given the stated similarities, there are grounds to suspect that the mechanisms of expansion are identical in mice and humans, only occurring at an accelerated pace in the mouse on account of the long repeat tract. In this case, the mouse model would function as a good model for human cases with mid-life age of onset as well. However, periodicity has not previously been reported in HD patients. A possible explanation could be that the repeat length in the R6/1 mouse is longer than the repeat length that has been analyzed in any HD human tissue with regard to somatic instability. The genomic localization of the randomly integrated HD gene fragment in R6/1 mice might modulate the CAG stability. Furthermore, CAG instability in HD patient brain cannot be analyzed at an early time-point that would allow for a direct comparison to the R6/1 data.

Importantly, CAG repeat expansion in human cortex is associated with an earlier age of disease onset, in addition to the role of the constitutional CAG repeat length [6]. This implies that there are disease modifiers that influence somatic instability, and conversely, factors that determine somatic instability which may modify disease pathogenesis. It is therefore critical to understand the mechanism of the different expansion processes and the factors involved.

To summarize, we present two different mechanisms of somatic CAG repeat expansion; a continuous bi-directional expansion in tail, lung, heart and spleen tissues, and a dramatic periodic expansion centred around 7-repeat insertions in striatum and cortex. Further experiments are needed to determine whether the 7-repeat step-size is independent of CAG tract length and species and it remains to be shown whether these two models can explain the expansions observed in other brain tissues and organs, as well as in humans. Nevertheless, these results provide significant new insights into *in vivo* expansion mechanisms, which may also be relevant to other triplet repeat disorders.

## Methods

### Ethics statement

All animal experiments have been approved by the local and national animal - and are carried out according to the regulation by FELASA (Federation of European Laboratory Animal Science Associations).

### Animals and tissues

B6CBA-Tg(HDexon1)61pb/J mice of the R6/1 line [54] with ∼119 CAG repeats in exon 1 of the *HTT* gene, were purchased from The Jackson Laboratories and either interbred or crossed with C57BL/6J *Fen1^E160D/E160D^* mice [36]. The mice were fed Rat and Mouse No.3 breeding diet (Special Diet Services) and tap water *ad libitum*. At 3 weeks of age the mice were anesthetized by i.p. injection of a combination of Midazolam (Dormicum “Roche”) and Fentanyl/Fluanisone (Hypnorm) solutions, and tail biopsies were taken. DNA from tail biopsies of the first two generations were lysed as described [36] and DNA isolated using standard NaCl precipitation or phenol/chloroform procedure. At 10 and 21 weeks of age the mice were sacrificed by cervical dislocation. The organs were harvested, frozen on dry ice and stored at −70°C. During dissection of striatum we lost 9 samples. DNA from all tissues and tails from the F2 and F3 generation was isolated according to the DNeasy Blood & Tissue kit (Qiagen GmbH, Germany).

### Genotyping

DNA from tail biopsies of 3-week old mice were used for genotyping. HD mice were genotyped with forward 5′-cggctgaggcagcagcggctgt-3′ and reverse 5′-gcagcagcagcagcaacagccgccaccgcc-3′ PCR primers [54] according to the Advantage GC 2 PCR Kit & Polymerase Mix (Clontech, CA). The *Fen1^+/+^* and/or *Fen1^E160D/E160D^* knock-in allele was genotyped as described [36].

### Sizing of CAG repeats

CAG repeats were sized by PCR with primers 5′-FAM-atgaaggccttcgagtccctcaagtccttc-3′ and 5′-ggcggctgaggaagctgagga-3′ according to [54] with slight modifications. Approximately 75ng of genomic DNA (this approximates to DNA extracts from ∼10,000 cells) was amplified with AmpliTaq Gold DNA polymerase with PCR Buffer II, 1.25 mM MgCl_2_ (Applied Biosystems, CA), and 2.5 mM dNTPs (GE Healthcare). The cycling conditions were 94°C for 10 min, 35 cycles of 94°C for 30 sec, 64°C for 30 sec, 72°C for 2 min, and a final extension at 72°C for 10 min. The FAM-labeled PCR products were mixed with GeneScan - 600 LIZ Size Standard and HiDi Formamide (Applied Biosystems) and run on an ABI 3730 Genetic Analyzer (Applied Biosystems). Sizing of the PCR fragments was performed by using the GeneMapper Software Version 3.7 (Applied Biosystems).

### Data analysis

All raw data was processed through a masked Nelder-Mead simplex fitting method, optimising free parameters of standard deviation, mean and amplitude to fit consecutive normal distributions sufficient to account for ≥98% of the total area of the raw data set. In the case of tail data, only a single normal distribution was required. These optimised parameters were returned as the means (μ), and standard deviations (σ), which were used to define the TNR lengths present in each data set. CAG repeat tracts were flanked by sequences 86 bp in length as verified by sequencing. Thus, the mean number of CAG triplets (μ_t_) present in a fragment analysis sample with a measured mean (μ_m_) is defined by μ_t_ = (μ_m_−86)/3. When analysing the periodicity present in striatum and cortex data, standard frequency analysis methods are not suitable, therefore our peak fitting method was used to fit consecutive normal distributions to the raw data (Figure 4A). We were unable to perform fitting analysis on 25 striatum samples due to the quality of the PCR product. The means (μ_a_, μ_b_ etc) and relative areas (A_a_, A_b_ etc) of each peak were calculated (Figure 4B). The intervals between neighbouring means (S_1_, S_2_ etc) were also recorded at both 10 and 21 weeks. The area (A) of each peak was used to estimate the number of cells containing triplet repeats that had expanded with a step size defined by the separation interval (S). The average area of the first peak in all 21-week data (A_a_ from Figure 4B) was used to estimate the proportion of non-expanding cells in the striatum as 54% (σ = 15.9), implying that approximately 45% of each striatum sample underwent periodic expansions within the first 21 weeks. By comparison, the proportion of dramatic expanding tissue observed in cortex samples was ∼20%. Previous work has shown that the dramatic expansion observed in the striatum of adult mouse brain tissue largely occur in the neuronal cells [15], [14], although slower expansion can be observed in glial cells and we used this to approximate the level of expansion in neuronal cells in combination with an estimate of the average number of expansion events that were measured in 21-week mice.

## Supporting Information

Figure S1 Further examples of curve-fits to raw data from striatum. We present a further set of raw fragment analysis curves paired immediately below with the corresponding normal distribution curve-fits to samples from 21-week striatum samples, in order to confirm the prevalence of the periodicity seen in the data. Individual normal distribution fits are shown in magenta, with the sum of all fitted curves shown in cyan.
(1.49 MB PDF)

Figure S2 Further examples of curve-fits to raw data from cortex. We present a further set of raw fragment analysis curves paired immediately below with the corresponding normal distribution curve-fits to samples from 21-week cortex samples, in order to confirm the prevalence of the periodicity seen in the data. Individual normal distribution fits are shown in magenta, with the sum of all fitted curves shown in cyan.
(0.49 MB PDF)

Figure S3 Comparison of 6-week striatum with 3-week tail. In order to further confirm that expansion peaks in striatum tissue develop with time and that the 3-week tail sample is a legitimate representation of the condition of a given 3-week organ sample, we present the results from a single mouse which was sacrificed at 6 weeks of age. Here we show the results from the 3-week tail sample (A), to be compared with the striatum sample at 6-weeks with a mean of 122 repeats (B) and cortex with a main peak at 120 repeats and a small peak at 126 repeats (C). The striatum sample shows a mean slightly higher than the 3-week tail mean. However, the limitations of the analysis technique mean that the distribution could be similar to that shown for cortex, with two peaks which are bundled together by the curve-fitting algorithm. To maintain the unbiased nature of the analysis, it is necessary to admit the uncertainly and refrain from attempting guided curve-fitting. Irrespective of this, the small disparity between the 6-week striatum and 3-week tail mean values supports our stated assumption that the near-birth repeat level in all organs is well approximated by the level measured in a 3-week tail biopsy.
(0.24 MB PDF)

Figure S4 Proportions present in samples with mixed populations. Our ability to estimate the proportions present in samples with mixed populations of repeat lengths was tested, as shown below. Two separate samples containing 120 and 129 repeats were mixed in a variety of ratios, and then processed by fragment analysis. The spectra were analyzed as described in the paper and the fitted curves are shown below. The results show that the technique is more than adequate for the estimation of sample distributions, particularly when applied to an entire data set.
(0.27 MB PDF)

Figure S5 Striatum samples separated by repeat length after three consecutive 10× dilutions. To clarify that individual fragment analysis samples from striatum are composed of DNA, we decided to perform a limited set of serial dilutions on a single 21-week striatum sample and then proceed with fragment analysis, in order to see whether the original sample could be divided up into individual peaks, thereby further validating our multiple curve-fitting analysis method. A) Fragments from an original sample showing separable peaks (black below) were subjected to 3 successive 10× dilutions. This resulted in a group of samples in which there was a finite chance that fragments of only one, or a few, different lengths would exist, compared to the original sample. Several of these samples were then amplified by PCR and the peaks from three separate samples are shown here (blue, green and red), with clearly discernable means that correlate with the peaks shown in the original data. These results show that where individual length fragments are separated out by dilution, their means align well with the peaks that are visible in the original data, reinforcing the conclusion that striatum samples contain TNR tracts which have expanded periodically by multiples of ∼7 insertions. While a small pool PCR (spPCR) technique has been used to show that the fragment analysis curve resembles the distribution of individual fragment lengths measured in small pools (Gonitel et al. DNA instability in postmitotic neurons. ProcNatlAcadSci USA (2008) vol. 105 (9) pp. 3467–72 Figure S10), the resolution of fragment lengths detected is too low to detect the periodicity we show here. We show that individual peaks that align with the peaks found in the original samples at 10 weeks (B) and 21 weeks (C) can be found at a range of dilutions. What is notable here is that the standard deviation of these peaks appears to be fairly independent of the dilution level. At the highest dilutions, the expectation is that the PCR amplification occurs from a single fragment, while lower dilutions would be expected to start with a greater number of fragments. The consistency of standard deviations suggests a high similarity between all fragments in the less dilute samples. A credible high-resolution spPCR approach would unfortunately require in the region of thousands of samples before it improved upon the results from the existing fragment analysis technique.
(1.14 MB PDF)

Figure S6 Simulation of periodic expansion with and without slipped-strand expansion. Here we present the results of three simulations of 7-repeat periodic expansion in a population of 20,000 cells, with varying levels of simultaneous slipped-strand expansion and contraction as observed in tail. In all cases the probability of a 7-repeat step, a unitary expansion and a unitary contraction are shown. In the first case (A), no slipped strand expansion and contraction are allowed, leaving clearly defined peaks throughout the data, similar to those observed in real striatum data. (B) is simulated with approximately a quarter of the level of slipped strand expansion and contraction, which renders the peaks indistinguishable. In (C) with the measured levels of expansion and contraction in tail, the distribution becomes utterly uninformative. This is the basis for our argument that slipped-strand and periodic expansions do not occur simultaneously in striatum.
(0.11 MB PDF)

Figure S7 Increased replication does not increase expansion rate in spleen. Proliferating cell nuclear antigen staining of frozen spleen sections from HD mice with Fen1 mutation (lymphoma) and without Fen1 mutation (normal spleen) are shown (A). Histograms of expansion levels in 21-week spleen samples presenting lymphomas from HD mice with Fen1 mutation (blue) and normal spleens from HD mice (red), show no significant difference (B). Increased replication in lymphoma tissues does not affect the rate of CAG repeat expansion. The continuous expansion in spleen therefore appears to be independent of replication.
(1.78 MB PDF)

Figure S8 Increased replication does not increase expansion rate in spleen. (A) A proposed model mechanism for the slight expansions measured in CAG tracts of tail tissue is shown. Since this expansion is the result of a multitude of small expansion and contraction events within individual cells, we present a model that accounts for both processes by alternate processing of migrated loop-outs formed as slipped-strand structures within the repeat sequence. Initiated by a bubble, loop-out, or cruciform structure, the loops on opposite strands may migrate apart, rather than resolving back into a duplex formation. Should a loop be processed as an error by either of the alternate mechanisms shown, a single contraction or expansion event can occur. Removal of a loop structure on either strand causes a single repeat contraction, while nicking and gap-filling on the strand opposite to a loop results in a single repeat expansion. A slight bias favouring expansion over contraction will result in the overall population expansion measured in tissue samples. (B) Explanation of simulations videos: All videos show the starting 3-week distribution (blue) and the daily progress of the distribution under the given expansion mechanism, up to 21-weeks (red). Additionally, for extra clarity, inset on the right is a matrix of 10,000 points coloured by the number of repeats in each individual cell, to represent the actual number of repeats in each cell. Meanwhile, inset on the left is a histogram of the probabilities for expansion and contraction for each CAG repeat insert length, which have been chosen for the simulation.
(2.11 MB PDF)

Figure S9 Hypothetical hairpin-based model for periodic expansion. There are several potential models that could explain a periodic expansion mechanism with an average expansion length of 7 repeats. All of these require some inherent stabilisation of a loop of DNA, whether it be self-hybridizing, or in coordination with a protein or complex which interacts with DNA. In the following model we propose a means by which a hairpin can be stabilized around an average of 7 repeats in length. The hairpin formation should be considered as a Markov chain of transitions between states, whereby the mean/most-stable state is gives a 7-repeat hairpin and other lengths are distributed around this mean. A proposed pathway for periodic expansion by the step-wise insertion of 7-repeat hairpins is shown. After a preliminary strand break the CAG triplets are displaced from the DNA duplex and initially form an unordered flap. As the flap length increases, the flap alternates between metastable folded and disordered states. Continued strand displacement increases the overall length of the flap up to and beyond, 6 repeats. At this point, depending upon the instantaneous state of flap folding, there are effectively two possible folding pathways. The first pathway is shown to the left (blue background), whereby the first six repeats of the flap fold stably into a hairpin, which is eventually recognised as an erroneous structure and correctly repaired. There is no resulting expansion. A finitely probable alternative pathway is shown to the right. A stable 6-repeat hairpin is formed from CAG triplets 2 to 7 of the flap, leaving a single CAG repeat on the 5-end of the hairpin. The overhanging CAG triplet may hybridize with an unpaired CTG on the complementary strand, temporarily stabilizing the hairpin on the duplex. The stabilizing flap facilitates gap-filling repair and ligation of the CAG loop to the duplex DNA. Subsequent repair of a nick or a lesion along the CTG strand causes the extra CAG repeats to be spliced into the sequence, generating a single 7-repeat expansion event. Inevitably there will be more or less stable - and consequently likely - flap conformations with lengths greater or less than 7 repeats, but a mean-length or most probable length of 7-repeats would produce the observed expansions. While this may not be the only means of inserting a longer stabilized repeat sequence into the original sequence, the essential length-dependent stability of loopout structures which may also function as the basis for expansion would be related to the stability of the hairpins illustrated here.
(0.54 MB PDF)

Figure S10 Replicate PCR, striatum samples from the same mouse. In order to further confirm the periodicity we observe in our striatum samples, we have performed replicate PCR on 9 samples from the same mouse, which we show below. As is clear from the figures, periodicity is a consistent feature of the samples, although the size and mean position of the peaks show some variation between replicate samples. The variability between peak heights could be attributable to sampling error during the PCR preparation or one-repeat expansion or contraction early in the PCR cycles as described in Text S1 and Figure S12. While we begin with 75ng of template DNA, we are unable to verify that all of this is finally accessible for amplification. The variability between peak positions could result from technical as well as biological factors. It remains clear however that a general periodicity is present in repeat samples and that the general trend of the periodicity within the data is calculable when considered over many samples.
(1.35 MB PDF)

Figure S11 Examples of data from Heart, Spleen and Lung, matching the pattern shown in tail data. In order to confirm our statement in the manuscript that Heart (A), Spleen (B) and Lung (C) data all show similar monomodal distributions to Tail data, we have included examples of curves from these three tissue types.
(0.81 MB PDF)

Figure S12 PCR of repeat sequences and the observation of periodicity. (A) Simulated error-prone PCR from a single molecule. Starting with a single 119 repeat template, and plotting all PCR products (coloured by length) on equal sized areas, we can see the development of length variability in the population shown. See Text S1 for full discussion and implications. (B) Analysis of the efficiency of PCR on HD versus Neil1. See Text S1 for full discussion and implications. Quantifying the amount of product (using Kodak Molecular Imaging software) returned the raw data results shown for 10ng of 500bp standard, Neil1 and HD. (C) Brief illustration of the relative amounts of PCR product dependent upon both first cycle and general PCR efficiencies. (D) Periodicity visible in samples amplified from 250ng genomic DNA. As an additional element, to demonstrate that periodicity can also be observed in samples amplified from a significantly larger sample of genomic DNA, we present two examples from one striatum sample, where 250ng of genomic DNA has been amplified and where periodicity is also observable.
(1.97 MB PDF)

Text S1 A deeper discussion of model parameters and estimations.
(0.03 MB DOC)

Text S2 PCR of repeat sequences and the observation of periodicity.
(0.03 MB DOC)

Video S1 CAG expansion/contraction 1.
(1.42 MB MOV)

Video S2 CAG expansion/contraction 1 2 3.
(1.42 MB MOV)

Video S3 CAG expansion/contraction 3.
(1.38 MB MOV)

Video S4 CAG expansion 4 5 6 7 8 9 10.
(1.44 MB MOV)

Video S5 CAG expansion/contraction 5.
(1.39 MB MOV)

Video S6 CAG expansion/contraction 5 6 7 8 9.
(1.40 MB MOV)

Video S7 CAG expansion/contraction equal probability 5 6 7 8 9 10.
(1.62 MB MOV)

Video S8 CAG expansion/contraction 7.
(1.31 MB MOV)
