# Supplementary material for: Continuous and Periodic Expansion of CAG Repeats in Huntington's Disease R6/1 Mice
Source: PLoS Genet. 2010 Dec 9;6(12):e1001242. doi: 10.1371/journal.pgen.1001242 (PMC3000365; doi:10.1371/journal.pgen.1001242)

Figure S2: Further examples of curve-fits to raw data from cortex:

All data is shown between approximately 400 and 600 nucleotides on x-axis.

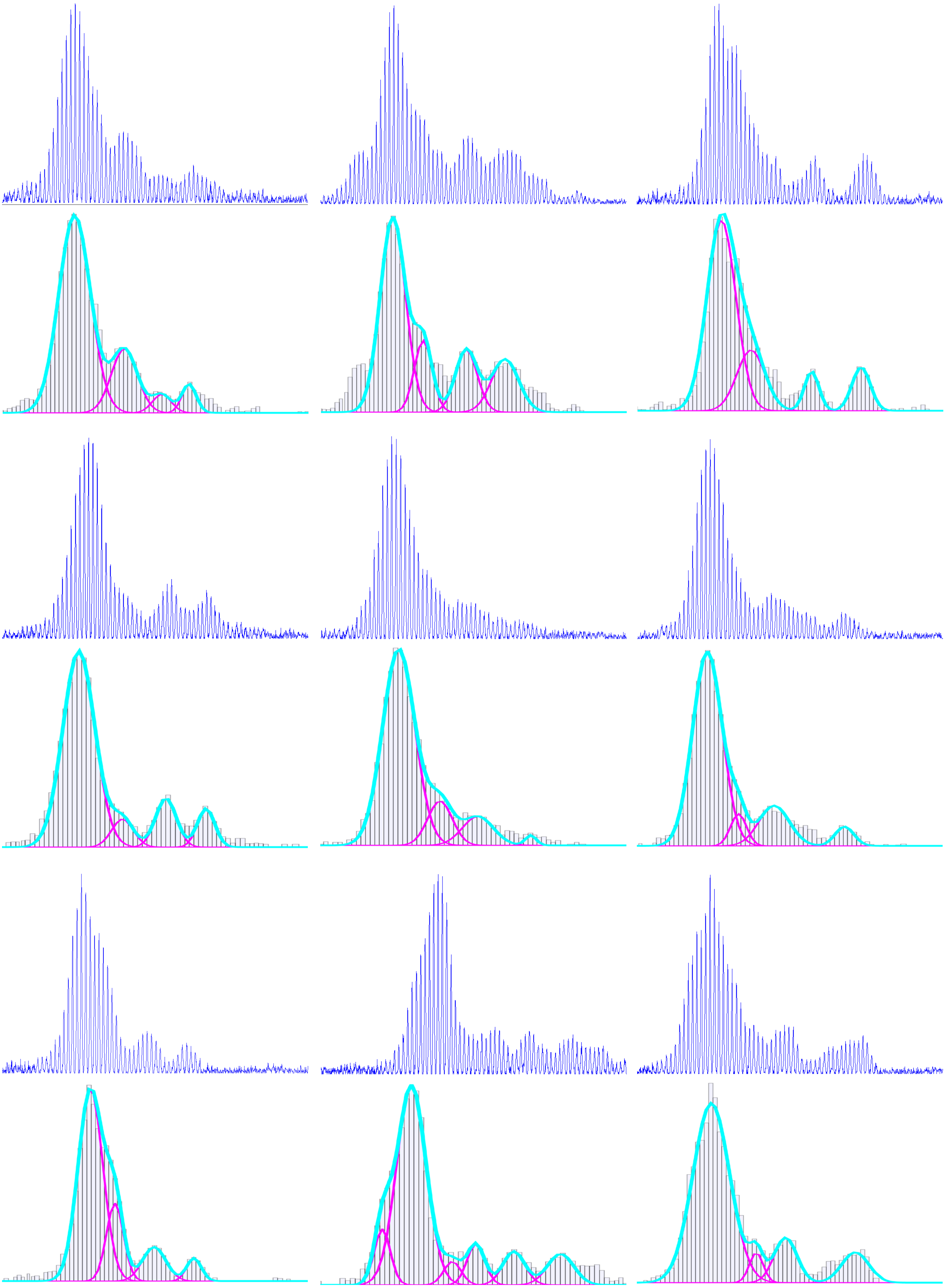

Supplement: Figure S2 — Further examples of curve-fits to raw data from cortex. We present a further set of raw fragment analysis curves paired immediately below with the corresponding normal distribution curve-fits to samples from 21-week cortex samples, in order to confirm the prevalence of the periodicity seen in the data. Individual normal distribution fits are shown in magenta, with the sum of all fitted curves shown in cyan. (0.49 MB PDF) [file pgen.1001242.s002.pdf]
