# Supplementary material for: Continuous and Periodic Expansion of CAG Repeats in Huntington's Disease R6/1 Mice
Source: PLoS Genet. 2010 Dec 9;6(12):e1001242. doi: 10.1371/journal.pgen.1001242 (PMC3000365; doi:10.1371/journal.pgen.1001242)

Figure S3: Comparison of 6-week striatum with 3-week tail:

A) Tail sample at 3-weeks with mean of 120 repeats

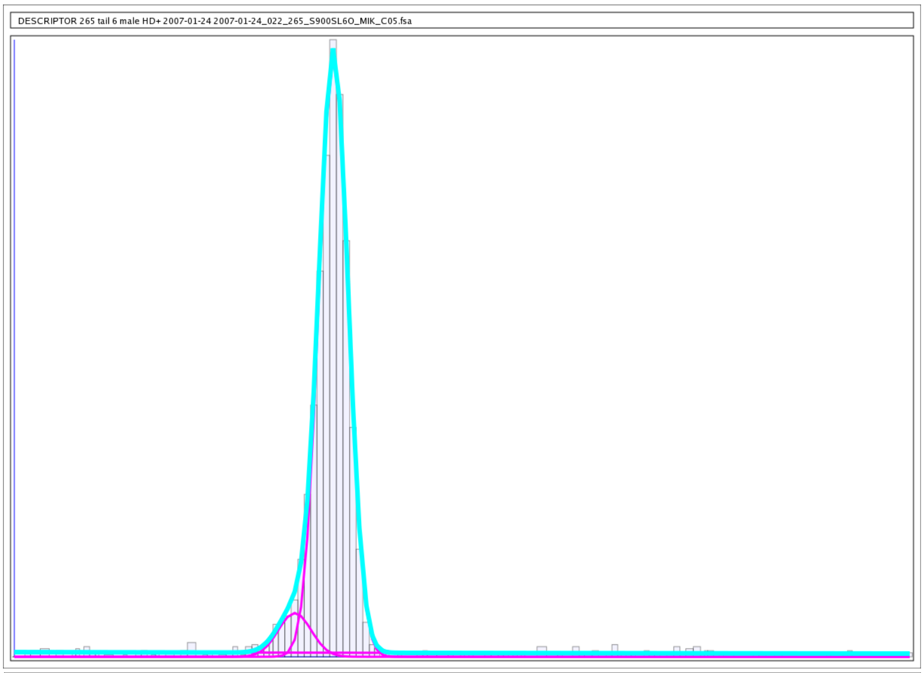

B) Striatum sample taken at 6-weeks

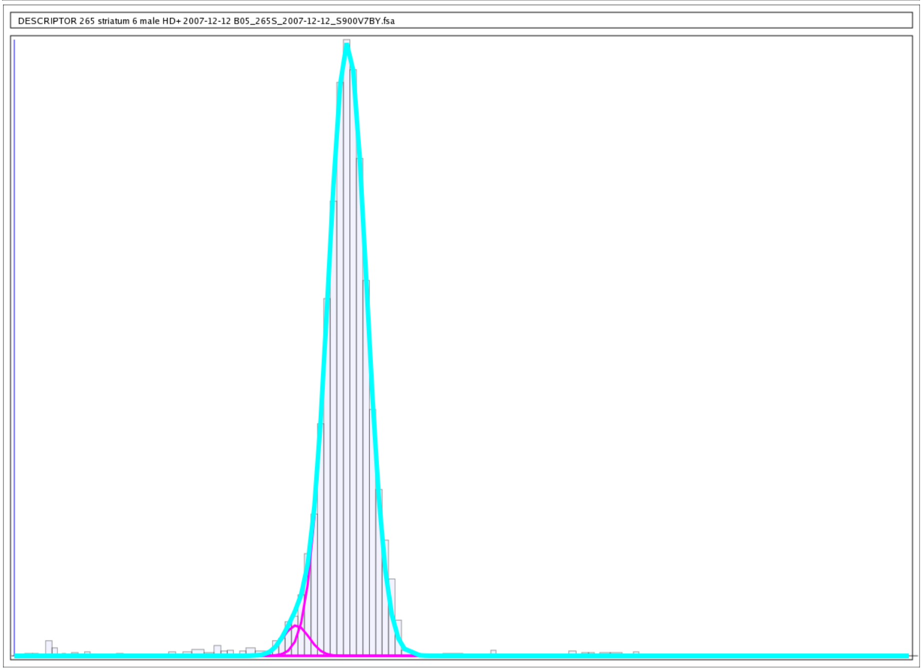

C) Cortex sample taken at 6-weeks

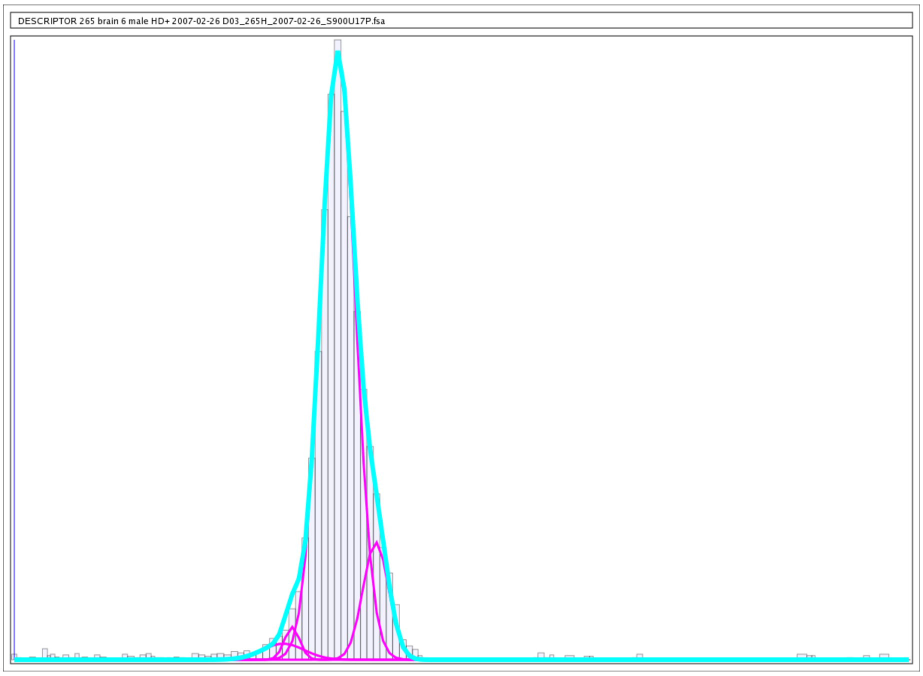

Supplement: Figure S3 — Comparison of 6-week striatum with 3-week tail. In order to further confirm that expansion peaks in striatum tissue develop with time and that the 3-week tail sample is a legitimate representation of the condition of a given 3-week organ sample, we present the results from a single mouse which was sacrificed at 6 weeks of age. Here we show the results from the 3-week tail sample (A), to be compared with the striatum sample at 6-weeks with a mean of 122 repeats (B) and cortex with a main peak at 120 repeats and a small peak at 126 repeats (C). The striatum sample shows a mean slightly higher than the 3-week tail mean. However, the limitations of the analysis technique mean that the distribution could be similar to that shown for cortex, with two peaks which are bundled together by the curve-fitting algorithm. To maintain the unbiased nature of the analysis, it is necessary to admit the uncertainly and refrain from attempting guided curve-fitting. Irrespective of this, the small disparity between the 6-week striatum and 3-week tail mean values supports our stated assumption that the near-birth repeat level in all organs is well approximated by the level measured in a 3-week tail biopsy. (0.24 MB PDF) [file pgen.1001242.s003.pdf]
