# Supplementary material for: Continuous and Periodic Expansion of CAG Repeats in Huntington's Disease R6/1 Mice
Source: PLoS Genet. 2010 Dec 9;6(12):e1001242. doi: 10.1371/journal.pgen.1001242 (PMC3000365; doi:10.1371/journal.pgen.1001242)
Supplement: Figure S4 — Proportions present in samples with mixed populations. Our ability to estimate the proportions present in samples with mixed populations of repeat lengths was tested, as shown below. Two separate samples containing 120 and 129 repeats were mixed in a variety of ratios, and then processed by fragment analysis. The spectra were analyzed as described in the paper and the fitted curves are shown below. The results show that the technique is more than adequate for the estimation of sample distributions, particularly when applied to an entire data set. (0.27 MB PDF) [file pgen.1001242.s004.pdf]

Figure S4: Proportions present in samples with mixed populations:

All ratios quoted as proportion of 120 TNRs : 129TNRs

Mixed ratio (%)		Measured ratio (%)
12.5 : 87.5	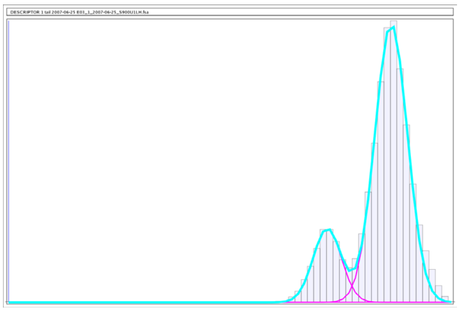	18.6 : 81.4
25.0 : 75.0	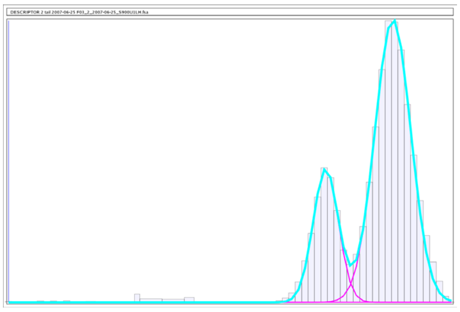	24.5 : 75.5
50.0 : 50.0	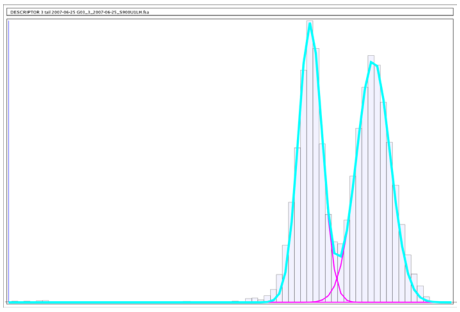	44.5 : 55.5
75.0 : 25.0	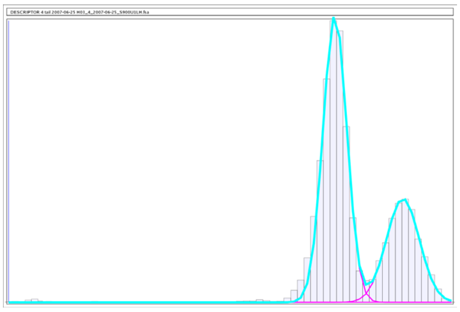	66.6 : 33.4
87.5 : 12.5	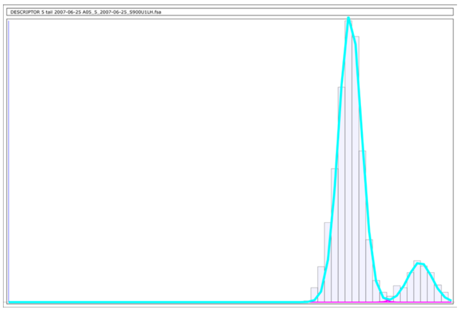	87.0 : 13.0
