# Supplementary figures and images for: Continuous and Periodic Expansion of CAG Repeats in Huntington's Disease R6/1 Mice
Source: PLoS Genet. 2010 Dec 9;6(12):e1001242. doi: 10.1371/journal.pgen.1001242 (PMC3000365; doi:10.1371/journal.pgen.1001242)

**Figure S5: Striatum samples separated by repeat length after three consecutive 10x dilutions:**

A

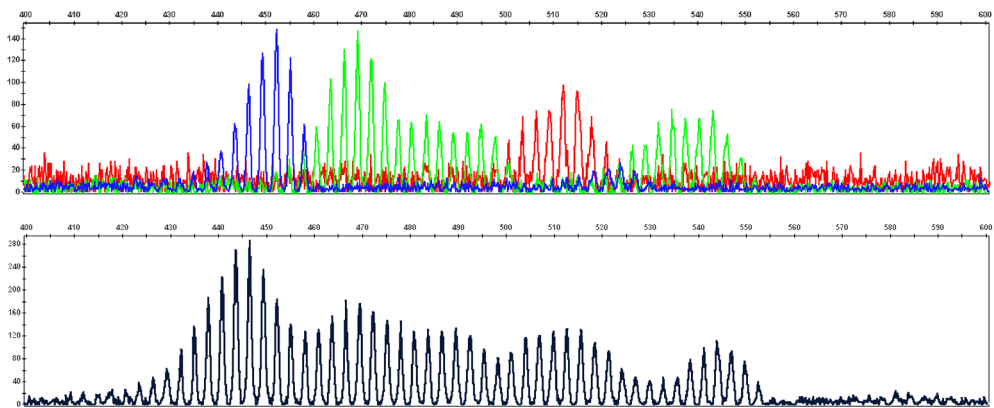

B

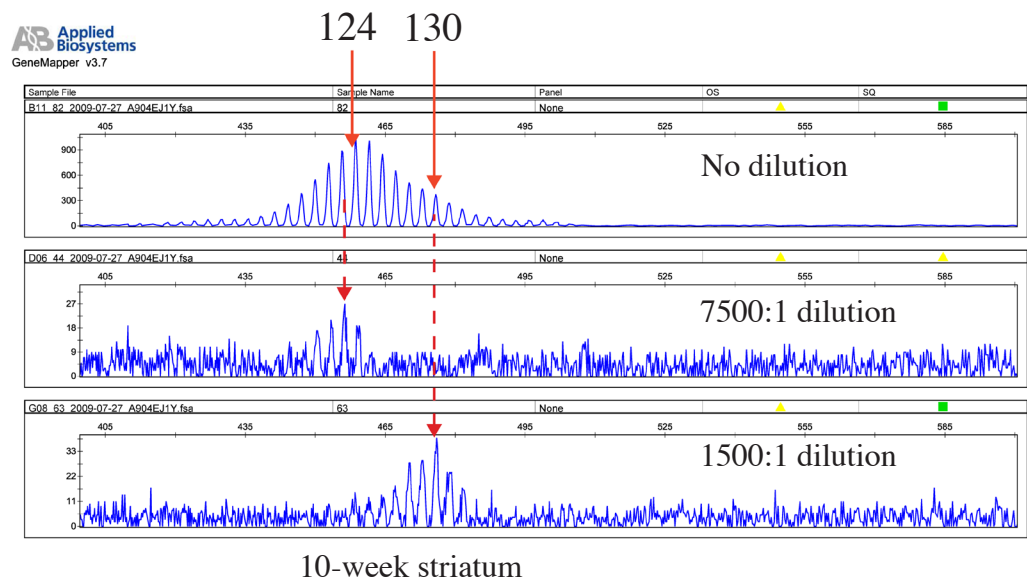

C

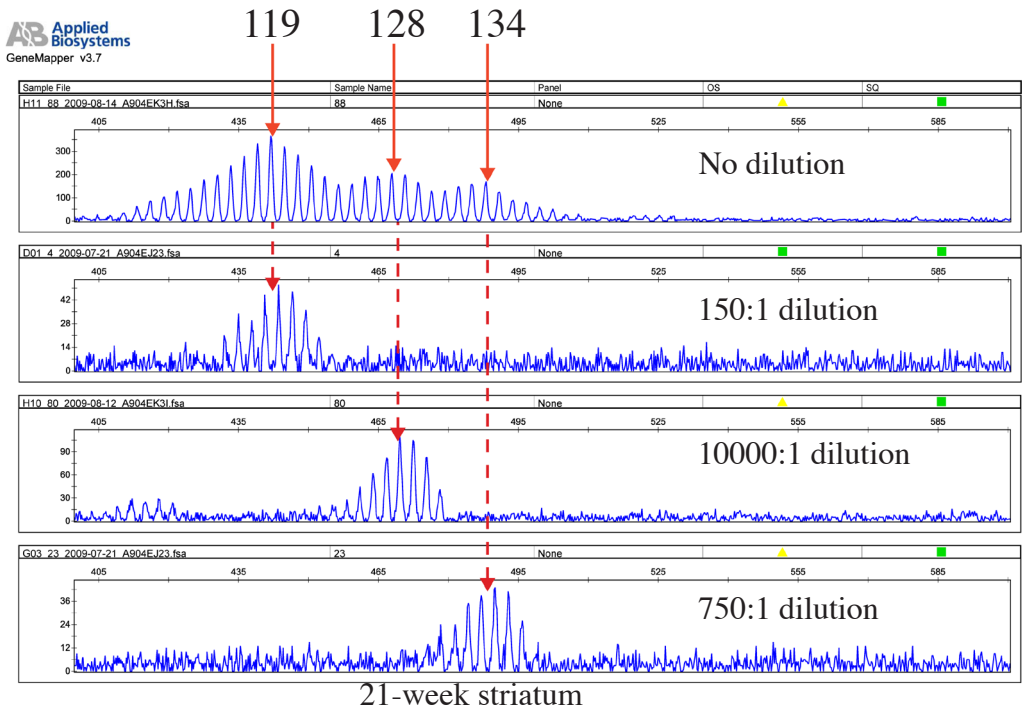

Supplement: Figure S5 — Striatum samples separated by repeat length after three consecutive 10× dilutions. To clarify that individual fragment analysis samples from striatum are composed of DNA, we decided to perform a limited set of serial dilutions on a single 21-week striatum sample and then proceed with fragment analysis, in order to see whether the original sample could be divided up into individual peaks, thereby further validating our multiple curve-fitting analysis method. A) Fragments from an original sample showing separable peaks (black below) were subjected to 3 successive 10× dilutions. This resulted in a group of samples in which there was a finite chance that fragments of only one, or a few, different lengths would exist, compared to the original sample. Several of these samples were then amplified by PCR and the peaks from three separate samples are shown here (blue, green and red), with clearly discernable means that correlate with the peaks shown in the original data. These results show that where individual length fragments are separated out by dilution, their means align well with the peaks that are visible in the original data, reinforcing the conclusion that striatum samples contain TNR tracts which have expanded periodically by multiples of ∼7 insertions. While a small pool PCR (spPCR) technique has been used to show that the fragment analysis curve resembles the distribution of individual fragment lengths measured in small pools (Gonitel et al. DNA instability in postmitotic neurons. ProcNatlAcadSci USA (2008) vol. 105 (9) pp. 3467–72 Figure S10), the resolution of fragment lengths detected is too low to detect the periodicity we show here. We show that individual peaks that align with the peaks found in the original samples at 10 weeks (B) and 21 weeks (C) can be found at a range of dilutions. What is notable here is that the standard deviation of these peaks appears to be fairly independent of the dilution level. At the highest dilutions, the expectation is that t [file pgen.1001242.s005.pdf]

**Figure S6: Simulation of periodic expansion with and without slipped-strand expansion**

**A**

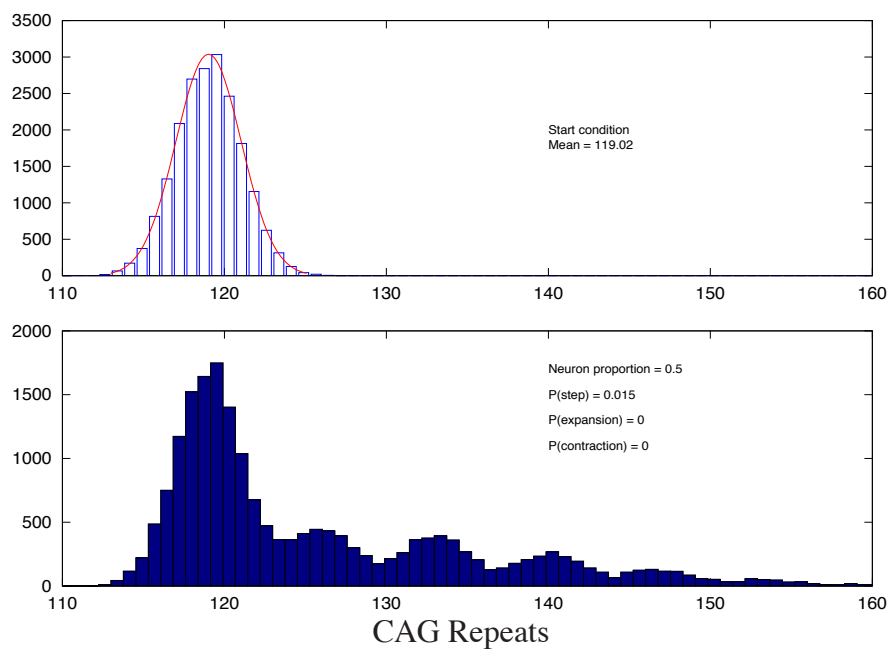

**B**

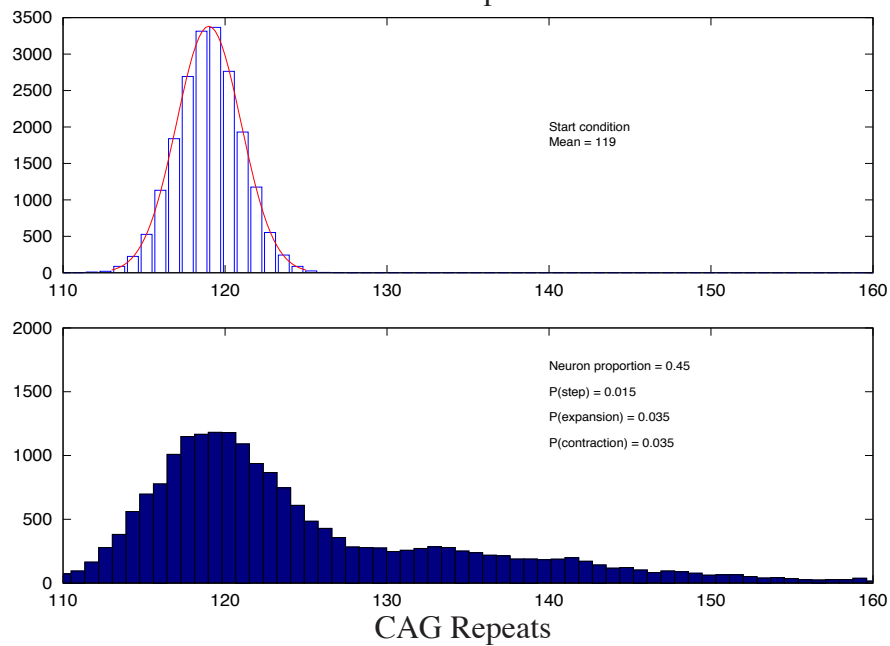

**C**

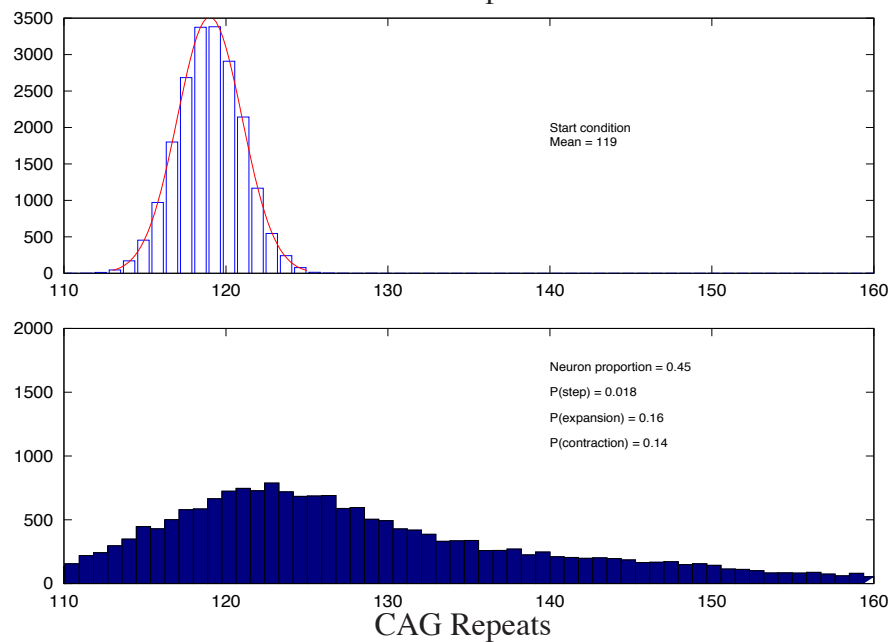

Supplement: Figure S6 — Simulation of periodic expansion with and without slipped-strand expansion. Here we present the results of three simulations of 7-repeat periodic expansion in a population of 20,000 cells, with varying levels of simultaneous slipped-strand expansion and contraction as observed in tail. In all cases the probability of a 7-repeat step, a unitary expansion and a unitary contraction are shown. In the first case (A), no slipped strand expansion and contraction are allowed, leaving clearly defined peaks throughout the data, similar to those observed in real striatum data. (B) is simulated with approximately a quarter of the level of slipped strand expansion and contraction, which renders the peaks indistinguishable. In (C) with the measured levels of expansion and contraction in tail, the distribution becomes utterly uninformative. This is the basis for our argument that slipped-strand and periodic expansions do not occur simultaneously in striatum. (0.11 MB PDF) [file pgen.1001242.s006.pdf]

**Figure S7: Increased replication does not increase expansion rate in spleen**

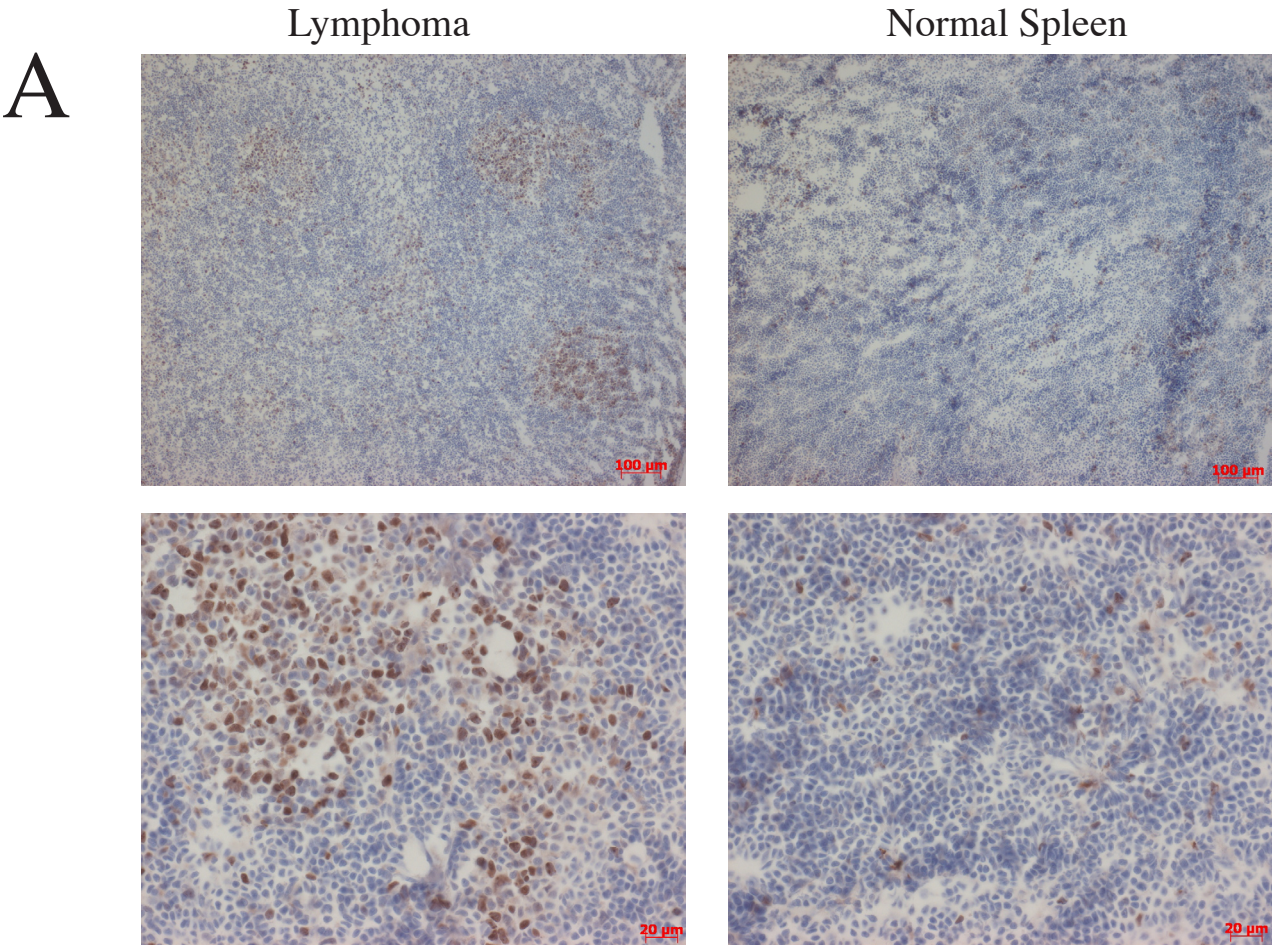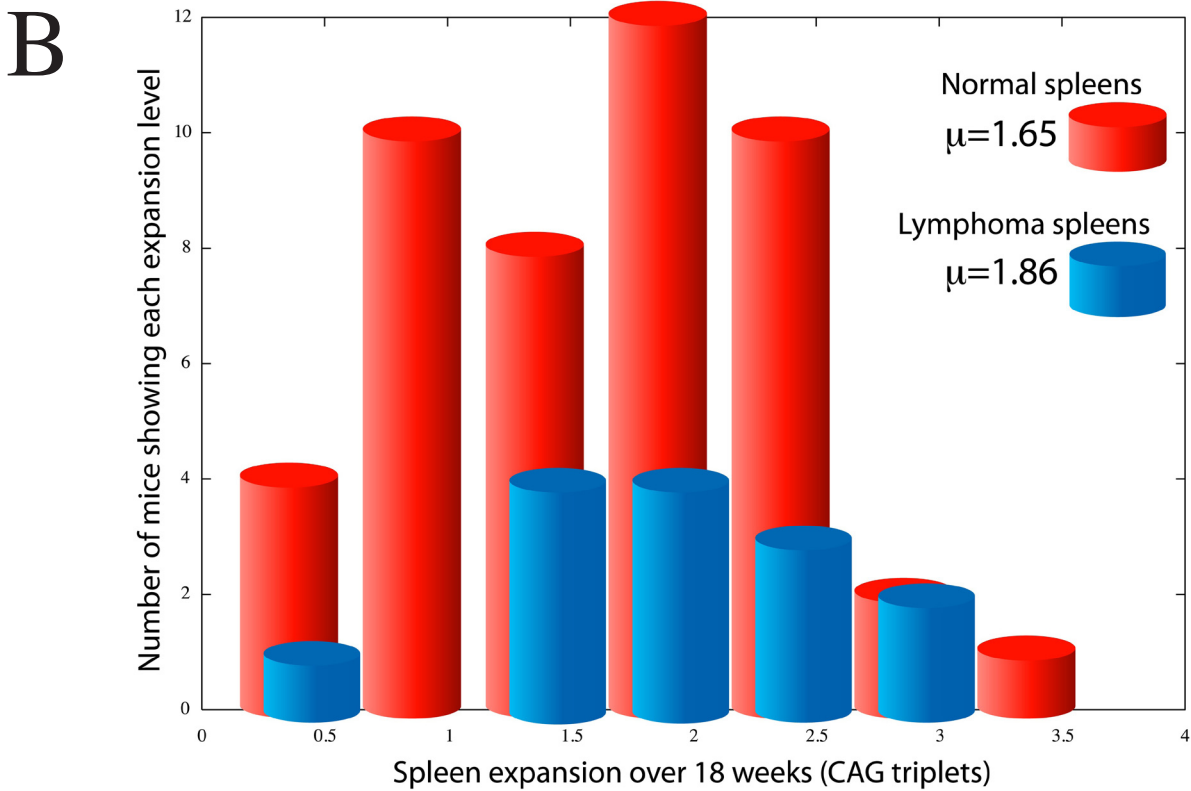

Supplement: Figure S7 — Increased replication does not increase expansion rate in spleen. Proliferating cell nuclear antigen staining of frozen spleen sections from HD mice with Fen1 mutation (lymphoma) and without Fen1 mutation (normal spleen) are shown (A). Histograms of expansion levels in 21-week spleen samples presenting lymphomas from HD mice with Fen1 mutation (blue) and normal spleens from HD mice (red), show no significant difference (B). Increased replication in lymphoma tissues does not affect the rate of CAG repeat expansion. The continuous expansion in spleen therefore appears to be independent of replication. (1.78 MB PDF) [file pgen.1001242.s007.pdf]

# Figure S9: Hypothetical hairpin-based model for periodic expansion

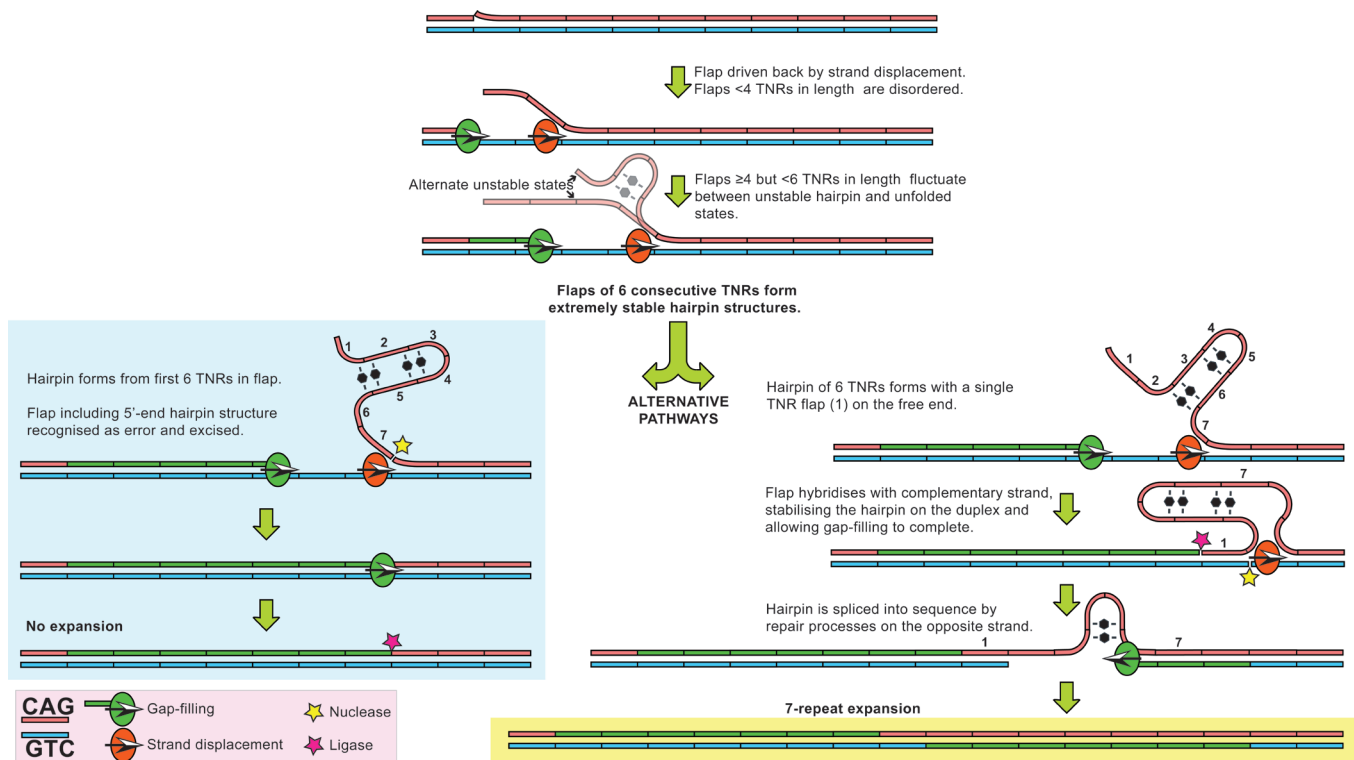

Supplement: Figure S9 — Hypothetical hairpin-based model for periodic expansion. There are several potential models that could explain a periodic expansion mechanism with an average expansion length of 7 repeats. All of these require some inherent stabilisation of a loop of DNA, whether it be self-hybridizing, or in coordination with a protein or complex which interacts with DNA. In the following model we propose a means by which a hairpin can be stabilized around an average of 7 repeats in length. The hairpin formation should be considered as a Markov chain of transitions between states, whereby the mean/most-stable state is gives a 7-repeat hairpin and other lengths are distributed around this mean. A proposed pathway for periodic expansion by the step-wise insertion of 7-repeat hairpins is shown. After a preliminary strand break the CAG triplets are displaced from the DNA duplex and initially form an unordered flap. As the flap length increases, the flap alternates between metastable folded and disordered states. Continued strand displacement increases the overall length of the flap up to and beyond, 6 repeats. At this point, depending upon the instantaneous state of flap folding, there are effectively two possible folding pathways. The first pathway is shown to the left (blue background), whereby the first six repeats of the flap fold stably into a hairpin, which is eventually recognised as an erroneous structure and correctly repaired. There is no resulting expansion. A finitely probable alternative pathway is shown to the right. A stable 6-repeat hairpin is formed from CAG triplets 2 to 7 of the flap, leaving a single CAG repeat on the 5-end of the hairpin. The overhanging CAG triplet may hybridize with an unpaired CTG on the complementary strand, temporarily stabilizing the hairpin on the duplex. The stabilizing flap facilitates gap-filling repair and ligation of the CAG loop to the duplex DNA. Subsequent repair of a nick or a lesion along the CTG strand causes the extra CAG repeats [file pgen.1001242.s009.pdf]

# Figure S10: Replicate PCR, striatum samples from the same mouse

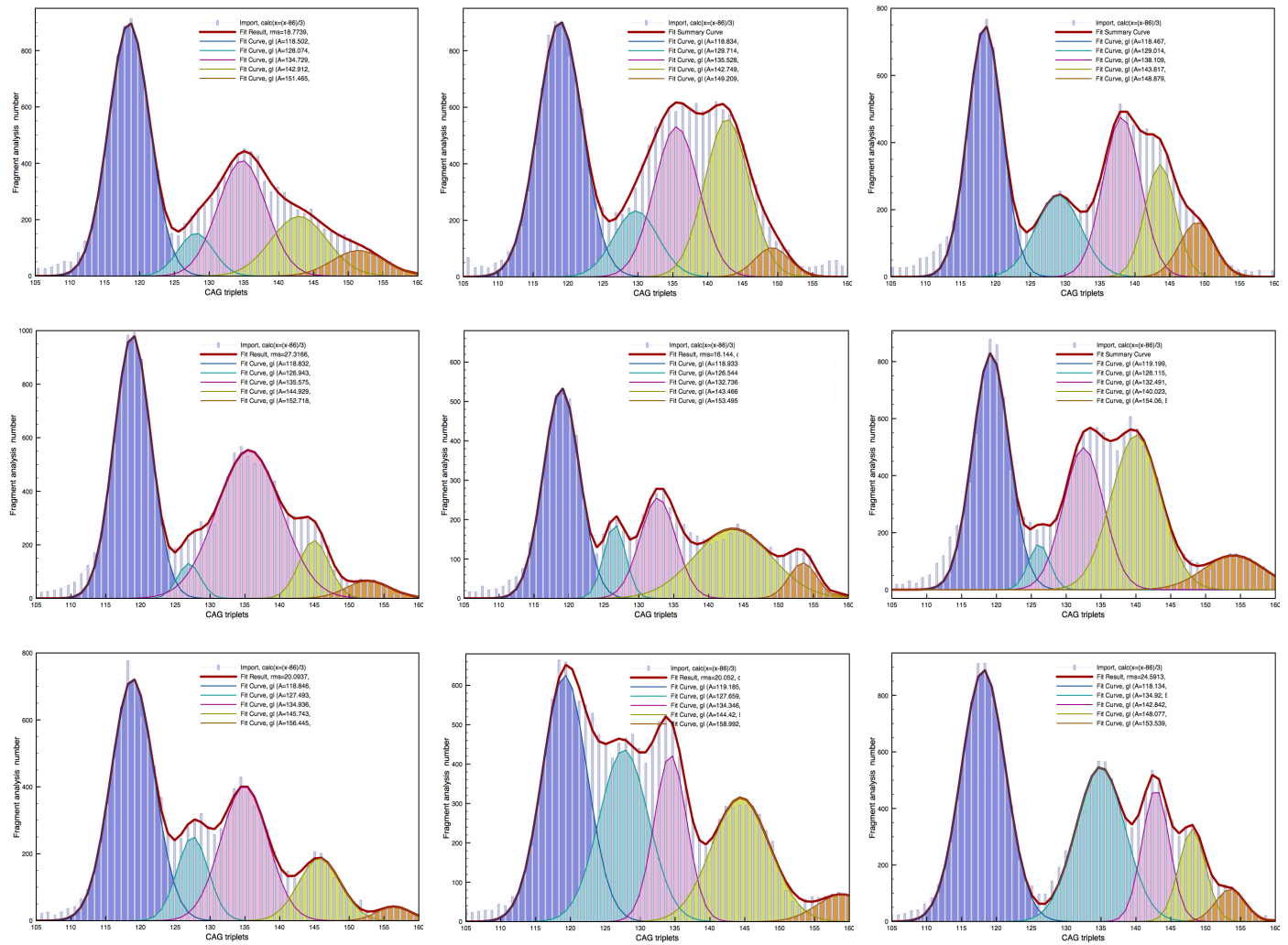

Supplement: Figure S10 — Replicate PCR, striatum samples from the same mouse. In order to further confirm the periodicity we observe in our striatum samples, we have performed replicate PCR on 9 samples from the same mouse, which we show below. As is clear from the figures, periodicity is a consistent feature of the samples, although the size and mean position of the peaks show some variation between replicate samples. The variability between peak heights could be attributable to sampling error during the PCR preparation or one-repeat expansion or contraction early in the PCR cycles as described in Text S1 and Figure S12. While we begin with 75ng of template DNA, we are unable to verify that all of this is finally accessible for amplification. The variability between peak positions could result from technical as well as biological factors. It remains clear however that a general periodicity is present in repeat samples and that the general trend of the periodicity within the data is calculable when considered over many samples. (1.35 MB PDF) [file pgen.1001242.s010.pdf]
