# Supplementary material for: Continuous and Periodic Expansion of CAG Repeats in Huntington's Disease R6/1 Mice
Source: PLoS Genet. 2010 Dec 9;6(12):e1001242. doi: 10.1371/journal.pgen.1001242 (PMC3000365; doi:10.1371/journal.pgen.1001242)

Figure S8: Model parameters and estimations for continuous expansion

A

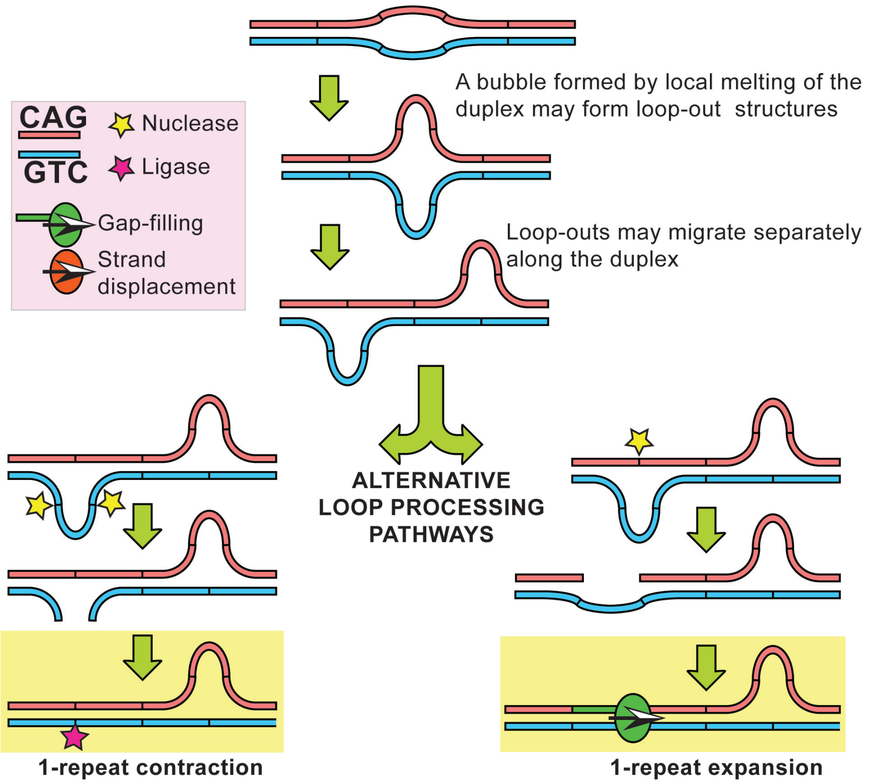

Model for continuous expansion by resolution of slipped-strand structures.

B

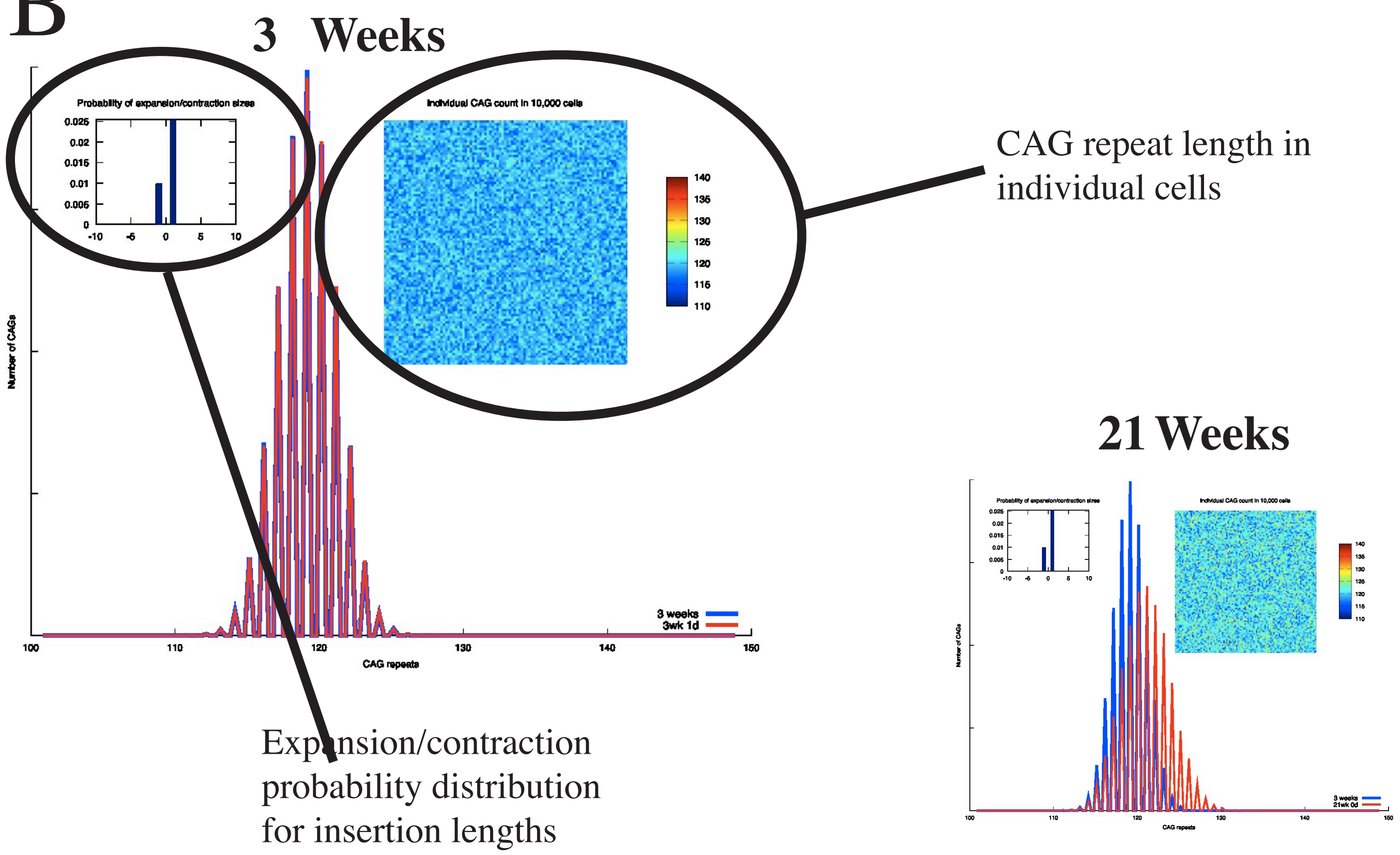

Supplement: Figure S8 — Increased replication does not increase expansion rate in spleen. (A) A proposed model mechanism for the slight expansions measured in CAG tracts of tail tissue is shown. Since this expansion is the result of a multitude of small expansion and contraction events within individual cells, we present a model that accounts for both processes by alternate processing of migrated loop-outs formed as slipped-strand structures within the repeat sequence. Initiated by a bubble, loop-out, or cruciform structure, the loops on opposite strands may migrate apart, rather than resolving back into a duplex formation. Should a loop be processed as an error by either of the alternate mechanisms shown, a single contraction or expansion event can occur. Removal of a loop structure on either strand causes a single repeat contraction, while nicking and gap-filling on the strand opposite to a loop results in a single repeat expansion. A slight bias favouring expansion over contraction will result in the overall population expansion measured in tissue samples. (B) Explanation of simulations videos: All videos show the starting 3-week distribution (blue) and the daily progress of the distribution under the given expansion mechanism, up to 21-weeks (red). Additionally, for extra clarity, inset on the right is a matrix of 10,000 points coloured by the number of repeats in each individual cell, to represent the actual number of repeats in each cell. Meanwhile, inset on the left is a histogram of the probabilities for expansion and contraction for each CAG repeat insert length, which have been chosen for the simulation. (2.11 MB PDF) [file pgen.1001242.s008.pdf]
