# Supplementary material for: Continuous and Periodic Expansion of CAG Repeats in Huntington's Disease R6/1 Mice
Source: PLoS Genet. 2010 Dec 9;6(12):e1001242. doi: 10.1371/journal.pgen.1001242 (PMC3000365; doi:10.1371/journal.pgen.1001242)

Figure S11: Examples of data from Heart, Spleen and Lung, matching the pattern shown in tail data.

A) HEART

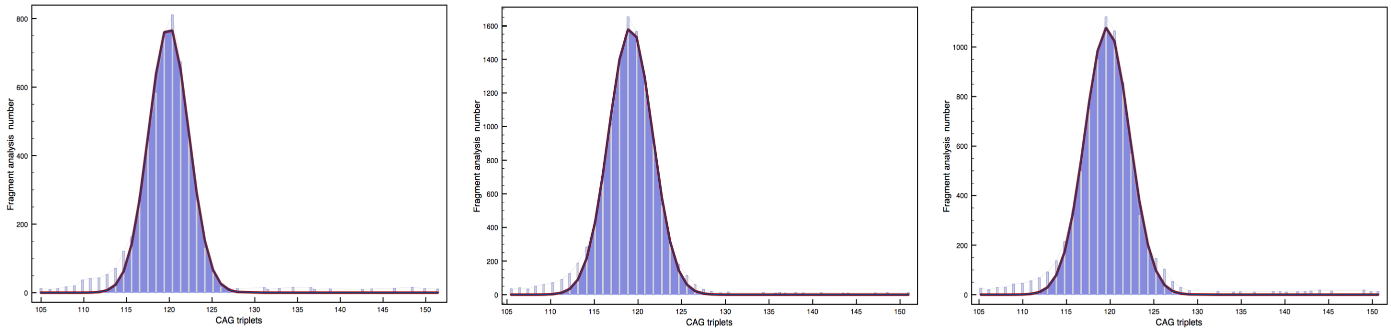

B) SPLEEN

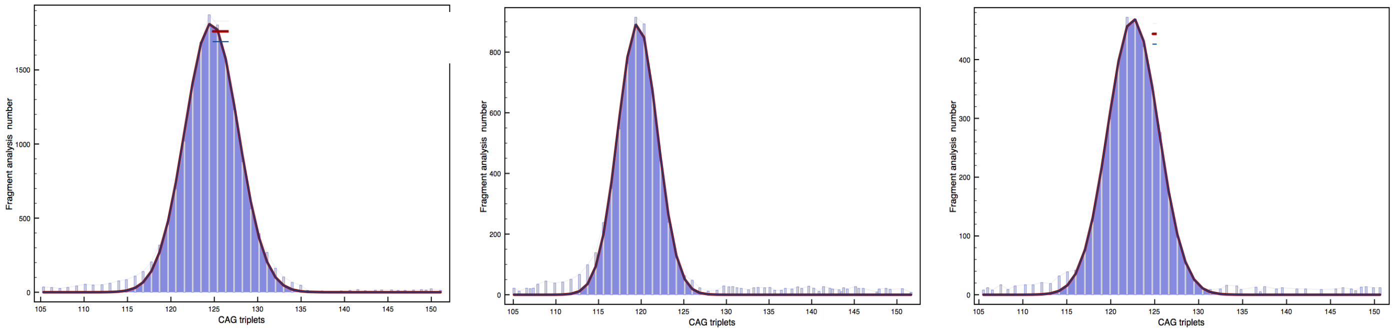

C) LUNG

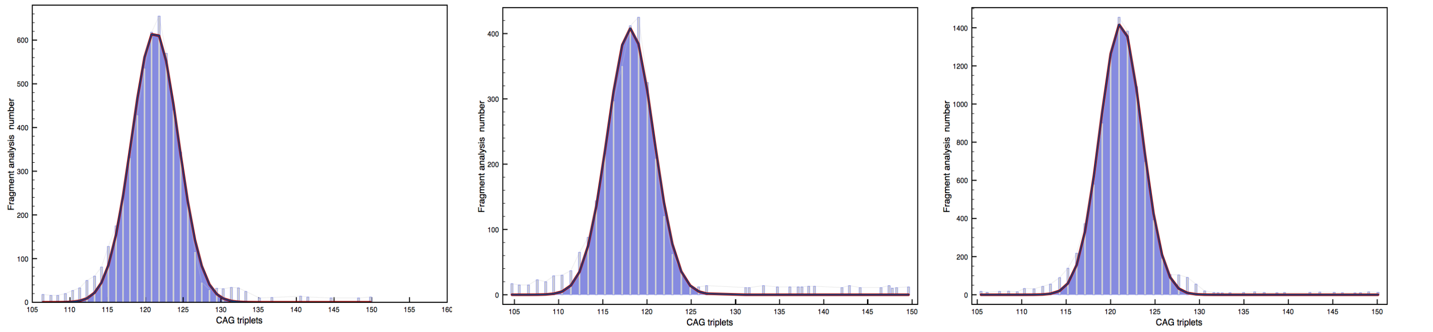

Supplement: Figure S11 — Examples of data from Heart, Spleen and Lung, matching the pattern shown in tail data. In order to confirm our statement in the manuscript that Heart (A), Spleen (B) and Lung (C) data all show similar monomodal distributions to Tail data, we have included examples of curves from these three tissue types. (0.81 MB PDF) [file pgen.1001242.s011.pdf]
