# Supplementary material for: Continuous and Periodic Expansion of CAG Repeats in Huntington's Disease R6/1 Mice
Source: PLoS Genet. 2010 Dec 9;6(12):e1001242. doi: 10.1371/journal.pgen.1001242 (PMC3000365; doi:10.1371/journal.pgen.1001242)
Supplement: Figure S12 — PCR of repeat sequences and the observation of periodicity. (A) Simulated error-prone PCR from a single molecule. Starting with a single 119 repeat template, and plotting all PCR products (coloured by length) on equal sized areas, we can see the development of length variability in the population shown. See Text S1 for full discussion and implications. (B) Analysis of the efficiency of PCR on HD versus Neil1. See Text S1 for full discussion and implications. Quantifying the amount of product (using Kodak Molecular Imaging software) returned the raw data results shown for 10ng of 500bp standard, Neil1 and HD. (C) Brief illustration of the relative amounts of PCR product dependent upon both first cycle and general PCR efficiencies. (D) Periodicity visible in samples amplified from 250ng genomic DNA. As an additional element, to demonstrate that periodicity can also be observed in samples amplified from a significantly larger sample of genomic DNA, we present two examples from one striatum sample, where 250ng of genomic DNA has been amplified and where periodicity is also observable. (1.97 MB PDF) [file pgen.1001242.s012.pdf]

Figure S12: PCR of repeat sequences and the observation of periodicity

A

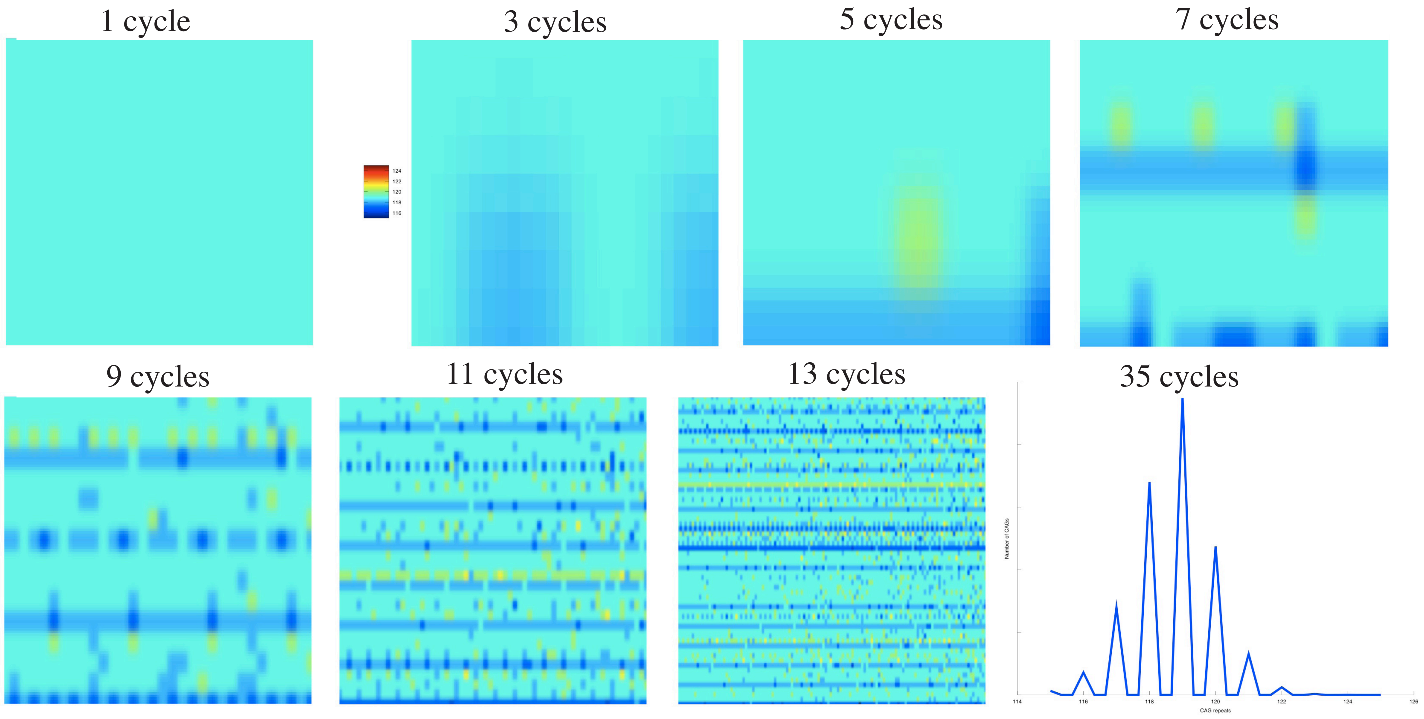

B

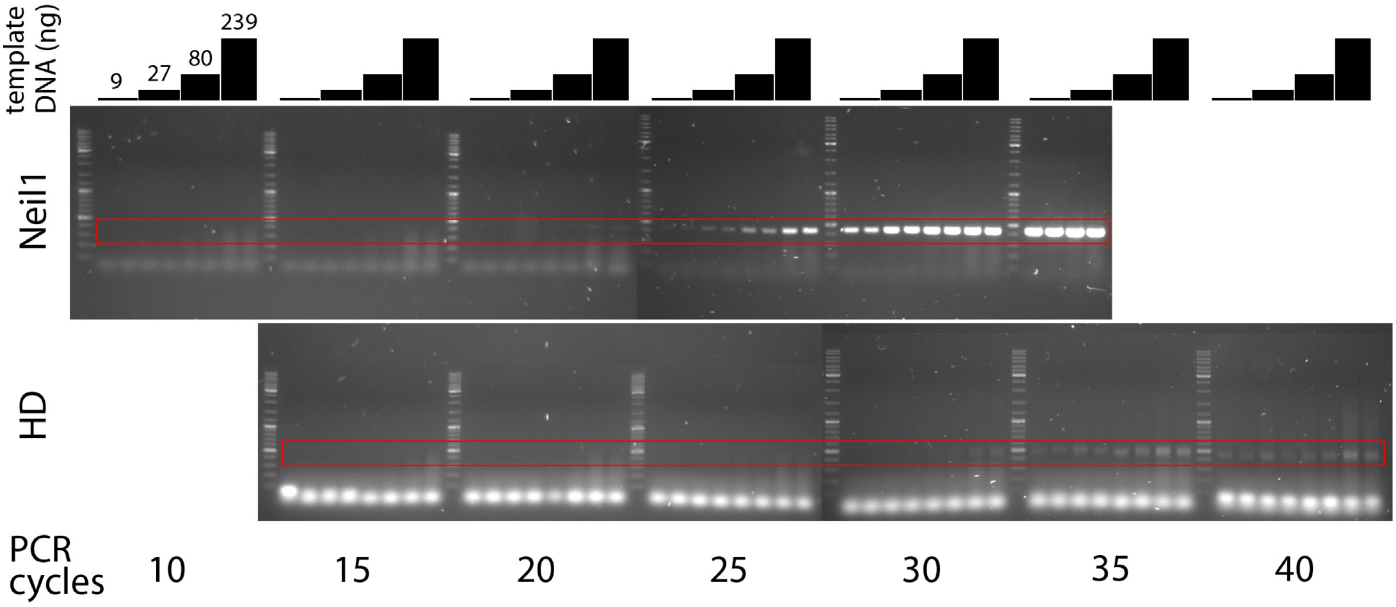

10ng standard	36701
10ng standard	37349
10ng standard	37738
10ng standard	38654
10ng standard	30337

Gene	PCR cycles	9	27	80	239
Neil1	35		198513		
Neil1	35		167489		
Neil1	35	152001			
Neil1	35	198538			
Neil1	30				191939
Neil1	30				182593
Neil1	30			187768	
Neil1	30			180594	
Neil1	30		173624		
Neil1	30		179325		
Neil1	30	121118			
Neil1	30	141585			
Neil1	25				134409
Neil1	25				125735
Neil1	25			51388	
Neil1	25			49542	
Neil1	25		17301		
Neil1	25		13615		
Neil1	25	3210			
Neil1	25	2540			
Neil1	20				15267
Neil1	20				14315
Neil1	20			5580	
Neil1	20			6398	

	PCR cycles	9	27	80	239
HD	40				26769
HD	40				37007
HD	40			14744	
HD	40			7654	
HD	40		7393		
HD	40		18805		
HD	40	11482			
HD	40	13274			
HD	35				33786
HD	35				33583
HD	35			23287	
HD	35			22341	
HD	35		11717		
HD	35		16284		
HD	35	9103			
HD	35	6392			
HD	30				13137
HD	30				8765
HD	30			3739	
HD	30			3009	

Raw data from analysis of gels

C

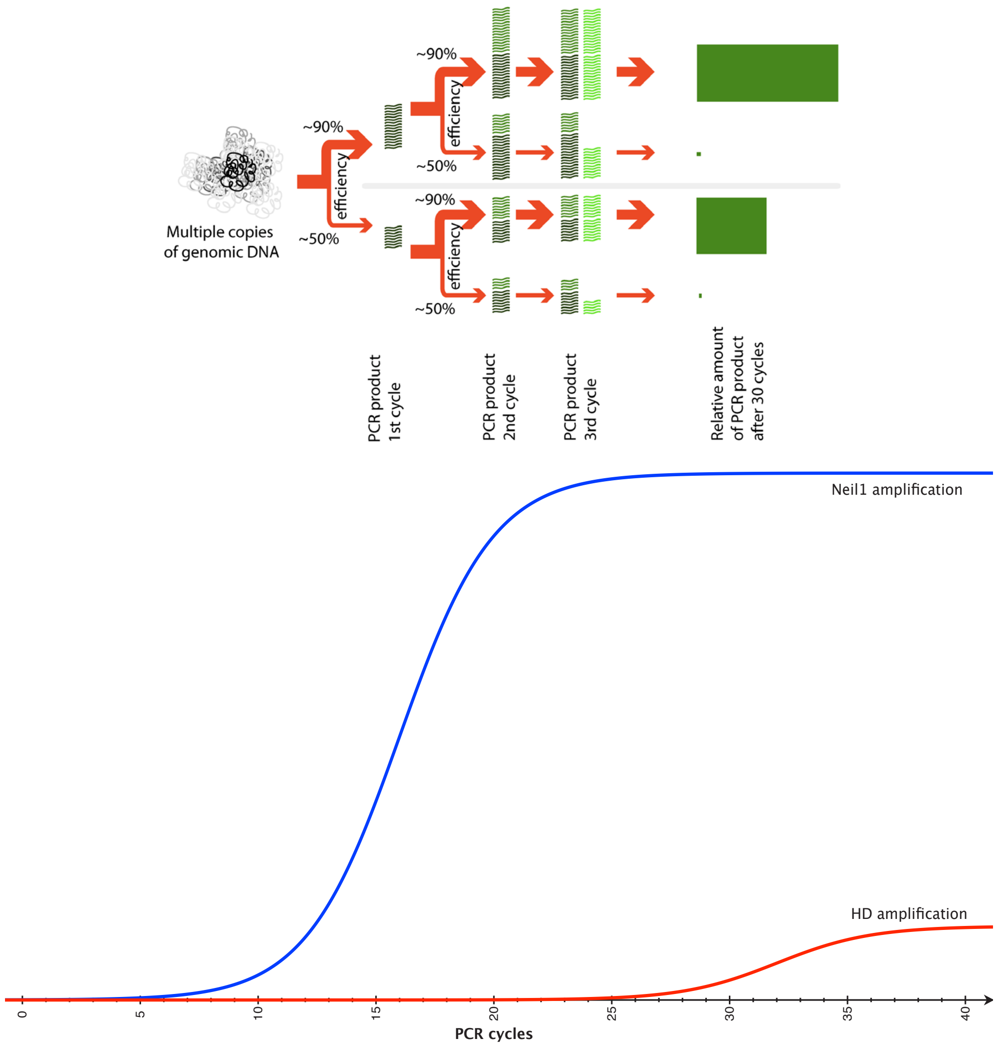

D

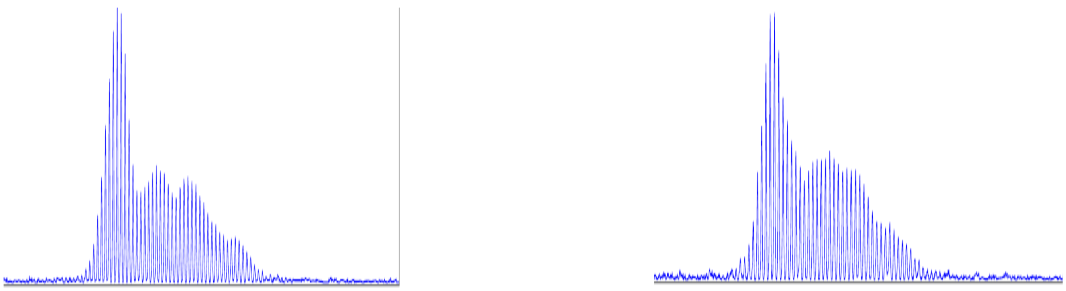

All data is shown between approximately 400 and 600 nucleotides on x-axis.
