# Supplementary material for: Continuous and Periodic Expansion of CAG Repeats in Huntington's Disease R6/1 Mice
Source: PLoS Genet. 2010 Dec 9;6(12):e1001242. doi: 10.1371/journal.pgen.1001242 (PMC3000365; doi:10.1371/journal.pgen.1001242)
Supplement: Text S1 — A deeper discussion of model parameters and estimations. (0.03 MB DOC) [file pgen.1001242.s013.doc]

Text S1

**A deeper discussion of model parameters and estimations**

**Continuous expansion:**

We raise the interesting possibility that single repeat expansions and contractions could be caused by a replication-independent process, based on the following estimations. Firstly we need to make a rough estimate for the amount of replication that occurs in mouse tail tissue between 3-weeks and 18-weeks of age. Generally quoted mouse body weights of 10g at 3 weeks and 30g (on average) when fully grown, imply a tripling in body weight during this period. Assuming that this is caused by a relatively even scaling-up of the whole body, would imply that the tail increases in volume by a factor of 3 in this period. Assuming further that individual tail cells are the same size at 3 weeks as at 21-weeks, would imply that the number of tail cells triples, presumably by a cell division process within these cells that requires replication (for a population of cells to triple in a given time, all cells must divide once and half of this total population must divide once more). If every replication event inserted 3 repeats into daughter cells, we could expect a similar distribution of CAG repeats to that we see for 21-week mice. However there are grounds to doubt that this is a consistent mechanism, not least because this would imply more rapid expansion in the earliest stages of development, when body size doubles repeatedly; but we do not see this when we compare the 3-week measurement to the number of repeats inherited from the parent generation. Furthermore, as discussed in Supporting Figure S7, increased levels of replication seen in spleen tissues with lymphomas did not correlate with an increased number of CAG repeats.

We emphasize here that we are not attempting to dismiss a role for replication in CAG expansion, however we feel that the above points are sufficient to warrant a discussion of potential alternative or concurrent mechanisms.

With regard to the thermodynamic feasibility of small loop-out structures, we acknowledge that small loop-outs are not thermodynamically stable enough to form and persist over a long time-period, but this does not affect their ability to form spontaneously. We contend that the appearance of segments of repeat sequences which melt and re-anneal in an out-of register manner, is - as postulated in Figure 5 of Gomes-Pereira et al. (Pms2 is a genetic enhancer of trinucleotide CAG.CTG repeat somatic mosaicism: implications for the mechanism of triplet repeat expansion. Hum Mol Genet (2004) vol. 13 (16) pp. 1815-25) - a rapid and volatile process. Single molecule biophysical experiments performed on repeat sequences have also shown that complementary triplet strands can slip reversibly against one another on sub-second timescales, even under tension (Kuhner et al. Force-induced DNA slippage. Biophysical Journal (2007) vol. 92 (7) pp. 2491-7) indicating high local rates of melting, diffusion and rehybridisation. Potential coincidence with the action of duplex-interacting proteins (for instance during transcription) may be sufficient to stabilize some of these short loop-outs as separate single-strand loops for long enough to allow them to be repaired as separate units. This forms the basis of our postulated model for continuous expansion, shown as a Figure S8-A.

**Potential corrections when estimating the rate of continuous expansion:**

We can make a relatively simplistic correction to our model based on the relative widths of the hedgehog distributions we observe at 3 weeks and at high dilutions, where we expect that the distribution has developed from a single CAG repeat tract.

Looking at Figure S5 we see that the hedgehog distributions generally span between 5 and 7 repeats (points distinctly above background noise), while an examination of the 3-week curves from figure 1 in the main text shows hedgehog distributions spanning 11 to 13-repeats. On the assumption that the standard deviations of the originating tail sample and the amplification process are additive, this would suggest that approximately half of the measured standard deviation in tail is due to the amplification process. This fixed value (3wk s.d. = 1.98 => amplification s.d. ~1) can then be subtracted from the 21-week standard deviation (21wk s.d. = 2.89 => sample s.d. of 1.89 repeats). Solving for p_e_ and p_c_ with these values gives a probability of expansion of 0.0183 and contraction probability of 0.0027 per day.

This is close enough to the estimated average expansion probability in striatum tissue (notwithstanding the possibilities for under- and over-estimation we mention in the manuscript) to raise the question as to whether the expansion is initiated in the same way. This we cannot answer, but we can still conclude firmly that (as we will shortly show here by simulation) the continuous expansion is based around short insertions with a length tending towards 1 repeat, while periodic expansion requires insertions with an average length of 7 repeats.

It is worth reiterating at this point that these models are an attempt to rationalise, parameterize and discuss the change in the distribution of repeat lengths that we have observed (the central feature of the article) in HD model mice, rather than a statement of absolute certainty as to the molecular mechanisms at work.

**Simulation of expansion modes: what we can show.**

We will begin by dealing with the choice of conditions tested, in regard to the simulation of continuous expansion. We have shown figures in the manuscript for a few sets of conditions in order to demonstrate the effect of differing probabilities on the model presented. However it is worth noting that the variables for pe and pc, which correctly match our observations, are the unique solutions to the two equations shown, in the article. In this case therefore, there is only one set of parameters that can produce the observed conditions, given the applied model. This moves on to the next point, as regards the applicability of the model. In the following text and associated video simulations, we discuss the effect of insert size upon the change in CAG distributions with time. The essence of this discussion is that as the insert size becomes progressively larger, the distribution tends to develop less normally. It is clear that with 5 CAG (Video S5) or 7 CAG (Video S8) inserts that the distribution develops “shoulders” (and later multimodality) rather than persisting as a broadening normal distribution. With inserts of 3 CAGs or less (Videos S1, S2 and S3), the distribution remains normal whilst the mean and standard deviation increase, no doubt in part due to the breadth of the original distribution. The implication of this is that the continuous expansion which occurs in tail must be caused by repetitive insertions and deletions of repeat sequences between 1 and 3 CAGs in length. Since no loop-out structure between 1 and 3 CAGs in length is obviously stable (without the opposite hairpins migrating a long way apart from each other) the probability of any one of these lengths being the insert length is most likely to be linked to the likelihood of initial formation for each loop-out structure. Since the probability of 3 adjacent bases melting simultaneously is significantly higher (roughly exponentially larger as a first approximation) than the probability of 6 adjacent bases melting simultaneously (and so on with increasing length), we assume that this correlates to the probability of formation for different length loop-out structures. Thus 1-repeat inserts would be more likely than 2-repeat inserts and so forth. Since the article is not aimed at a theoretical audience and is intended primarily to show the observed periodic expansion in brain tissues and contrast this with that seen in tail tissue, these details were left out of the discussion. They have been included here for the sake of completeness. As such, our model based solely on 1-repeat insertions and deletions provides an upper estimate of the rate of expansion and contraction activity.

In terms of a more general set of conditions for modeling the expansions observed in striatum, the observed periodicity and its persistence over several “steps” automatically limits us to a periodic insertion model, as discussed in the main article. The options for tuning a simulation of this are therefore limited to varying the probabilities of insert sizes grouped around the measured mean periodicity (in this case 7 repeats). In this case, given the breadth of the starting distribution and the level of measurement noise present, this is unable to contribute anything valuable in terms of parameterization.

Our conclusion regarding the periodic expansion seen in striatum is simply that the periodicity is centred around 7 CAG repeats, but we are unable to determine more details about the distribution of insert lengths around 7 repeats. This would imply that a 7-repeat flap is the most stable or likely conformation as a flap develops, however it does not preclude the existence of both shorter and longer hairpin structures. At most it suggests that shorter flap segments would not be so stable in a hairpin conformation and may preferentially melt and reform into longer more stable hairpins if the flap is long enough, before being incorporated into the duplex by repair. Meanwhile, if the length of a flap increases with time, it is reasonable to expect that the previously described process (whereby increasingly stable hairpins develop as the flap length increases) reaches a point where the hairpin becomes increasingly unlikely to melt and extend. While this again does not preclude the presence of longer hairpins, it points to the existence of an equilibrium condition with a “most likely” stable hairpin length, surrounded by less likely longer and shorter hairpin variants. It is this “most likely” length which we consider to be centred around 7-repeats. This is made clear by the simulation videos presented (Video S6 and Video S7). Equally, we cannot discount the possibility that the 7-repeat size could be the dominant size of looped-out structures which stabilize in the CAG repeat segments of striatum and cortex tissue, and are subsequently incorporated into the sequence.
