# Supplementary material for: Continuous and Periodic Expansion of CAG Repeats in Huntington's Disease R6/1 Mice
Source: PLoS Genet. 2010 Dec 9;6(12):e1001242. doi: 10.1371/journal.pgen.1001242 (PMC3000365; doi:10.1371/journal.pgen.1001242)
Supplement: Text S2 — PCR of repeat sequences and the observation of periodicity. (0.03 MB DOC) [file pgen.1001242.s014.doc]

Text S2

**PCR of repeat sequences and the observation of**

**periodicity**

The novelty of the observed periodicity presented in this article inevitably means that we need to clarify why it has not previously been observed, and show beyond reasonable doubt that the peaks observed in the data are not the product of experimental artifact.

To this end, we will present the following:

Figure S12 A) Discussion and model of the process of amplification from a single CAG-repeat-containing DNA template and the ramifications with regard to PCR.

We have already discussed (in Figures S5 and S8) the standard deviation present in the PCR product after amplification from a single genomic template, where we see that the hedgehog distributions generally span between 5 and 7 repeats (points distinctly above background noise). In order to attain this distribution, we infer that each amplification cycle must have the potential to insert or delete repeats, with a finite probability; thereby producing the longer and shorter repeat fragments which are distributed about the mean. To create a first order model representing this, we chose to allow each PCR cycle a finite probability of inserting or deleting a single CAG repeat relative to the template used. This indicates that a 10-12% probability of single repeat expansion or contraction per cycle can account for the broadening of the distribution from a single molecule starting point. This relatively significant probability of expansion or contraction means that there is an inherent chance that the mean value of the distribution can stray away from the starting value, if an expansion or contraction occurs early enough in the PCR cycles. This means that larger numbers of starting templates are likely to produce an increasingly smooth final distribution, masking periodicity, although we note that periodicity is also observable in some data where 250ng genomic DNA was amplified (see section D). Additionally, extra PCR cycles will also broaden individual distributions further, thereby smearing out periodicity.

Figure S12 B) Analysis of the efficiency of PCR on HD, compared with a parallel set of amplification data for Neil1, including quantitative estimates from PCR data to approximate the initial amount of genomic DNA present for amplification.

In order to quantify the efficiency of PCR, both in terms of the starting molecule amplification (first cycle) and the subsequent amplification, we ran parallel PCR on a set of 4 different DNA concentrations and over several different total cycles, for both Neil1 and HD. The resulting product is shown on the gels below. The bands of interest are outlined in red and the nearby 500bp band in the standard ladder contains 10ng DNA on each occasion. Quantifying the amount of product (using Kodak Molecular Imaging software) returned the raw data results shown for 10ng of 500bp standard, Neil1 and HD. These intensities can then be used to estimate the rate of production by PCR.

There are two crucial values that must be estimated. The first is the general rate of amplification, or amplification efficiency per cycle, which can be derived by comparing the ratio of products with similar start concentrations and different total cycles (this has to be done in the exponential phase of amplification in order to be meaningful). The second is to use the measured amplification efficiency to calculate the estimated number of DNA templates present after the first PCR cycle. This allows us to estimate the first cycle efficiency, which tells us how accessible the target sequence is within the genomic DNA. It is clear that in order to ensure that we are not subject to sampling errors, the first cycle efficiency should ideally be > 10%.

We illustrate briefly the relative amounts of PCR product dependent upon both first cycle and general PCR efficiencies in Figure S12 C. It is clear that Neil1 product is generated with high efficiency, while HD has a significantly lower efficiency of production. We also show (right) an approximation for the relative amplifications seen.

Comparison of the PCR product between 20 and 25 cycles for Neil1 shows an average of 90% efficiency (25 times increase over 5 cycles) which can then be used to estimate that something in the region of 10000 accessible DNA templates were present after the first PCR cycle. Comparison of the PCR product on HD is slightly more difficult, on account of the variation in intensity seen on the gels. One approach would be to take the average product from each pair of identical samples, but in order to minimise any chance for error or overestimation, we have chosen to use the largest change between neighbouring cycles, which in this case is a 7.74 times increase between 30 and 35 cycles with 80ng genomic DNA, which translates to a 51% PCR efficiency. Given an estimated 2.3x10^10^ PCR products present after 35 cycles on 80ng genomic DNA, a 51% amplification efficiency suggests that around >15000 accessible DNA templates are available after the first PCR cycle. This is far above the necessary 10% required to discount sampling error, given the presence of ~10000 initial copies of genomic DNA.
